# A *Plasmodium* Cross-Stage Antigen Contributes to the Development of Experimental Cerebral Malaria

**DOI:** 10.3389/fimmu.2018.01875

**Published:** 2018-08-14

**Authors:** Priyanka Fernandes, Shanshan W. Howland, Kirsten Heiss, Angelika Hoffmann, Maria A. Hernández-Castañeda, Klára Obrová, Roland Frank, Philipp Wiedemann, Martin Bendzus, Laurent Rénia, Ann-Kristin Mueller

**Affiliations:** ^1^Centre for Infectious Diseases, Parasitology Unit, University Hospital Heidelberg, Heidelberg, Germany; ^2^Singapore Immunology Network, Agency for Science, Technology and Research (A*STAR), Singapore, Singapore; ^3^German Centre for Infection Research (DZIF), Heidelberg, Germany; ^4^Department of Neuroradiology, Heidelberg University Hospital, Heidelberg, Germany; ^5^Division of Experimental Radiology, Department of Neuroradiology, Heidelberg University Hospital, Heidelberg, Germany; ^6^Department of Biotechnology, Mannheim University of Applied Sciences, Mannheim, Germany

**Keywords:** malaria, *Plasmodium berghei*, experimental cerebral malaria, pre-erythrocytic stages, CD8 T cells, cross-presentation

## Abstract

Cerebral malaria is a complex neurological syndrome caused by an infection with *Plasmodium falciparum* parasites and is exclusively attributed to a series of host–parasite interactions at the pathological blood-stage of infection. In contrast, the preceding intra-hepatic phase of replication is generally considered clinically silent and thereby excluded from playing any role in the development of neurological symptoms. In this study, however, we present an antigen *Pb*maLS_05 that is presented to the host immune system by both pre-erythrocytic and intra-erythrocytic stages and contributes to the development of cerebral malaria in mice. Although deletion of the endogenous *Pb*maLS_05 prevented the development of experimental cerebral malaria (ECM) in susceptible mice after both sporozoite and infected red blood cell (iRBC) infections, we observed significant differences in contribution of the host immune response between both modes of inoculation. Moreover, *Pb*maLS_05-specific CD8^+^ T cells contributed to the development of ECM after sporozoite but not iRBC-infection, suggesting that pre-erythrocytic antigens like *Pb*maLS_05 can also contribute to the development of cerebral symptoms. Our data thus highlight the importance of the natural route of infection in the study of ECM, with potential implications for vaccine and therapeutic strategies against malaria.

## Introduction

Cerebral malaria is a severe manifestation of an infection with *Plasmodium falciparum* parasites and a major cause of morbidity and mortality in developing countries (1). It is characterized by reduced cerebral blood flow caused by parasite accumulation in the brain and is accompanied by brain swelling, retinopathy, neurological sequelae, coma, and rapid death, if left untreated (2–4). Incidence of cerebral malaria is higher in children than adults, causing mortality associated with severe neuropathology while patients who recover are often left with permanent neurological damage (5–9).

The development of cerebral malaria is considered a multi-faceted process involving several host and parasite factors. Cytoadherence of infected erythrocytes (iRBCs) to the endothelium through interactions between parasite-derived surface proteins like *Pf*EMP-1 and endothelial cell receptors like ICAM-1, EPCR, and CD36 (10, 11) helps to prevent splenic clearance, but indirectly contributes to the vascular dysfunction observed in chronic malaria patients, particularly in the brains of those with cerebral malaria (12–16). Sequestered parasites obstruct blood vessels in the brain causing hypoxia and vasogenic edema (17), leading to activation of the endothelium (18, 19) and the recruitment of iRBCs, leukocytes, neutrophils, and macrophages (20, 21) which together contribute to local inflammation and breakdown of the blood–brain barrier (22–30). Thus, both sequestration and inflammation independently or in combination contribute to vascular leakage and neurological damage (31).

Numerous factors responsible for cerebral malaria have been identified from animal models such as infection of C57BL/6 mice with *Plasmodium berghei* ANKA which is commonly used to study cerebral malaria. This model reproduces several features of human cerebral malaria (HCM), such as convulsions, retinopathy, brain swelling, coma, and death (32–34) and is termed experimental cerebral malaria (ECM). Similar to HCM, ECM is an immune-mediated pathology that also relies on the sequestration of parasites in the brain (35, 36) with many studies attributing the development of ECM to interactions between the host spleen and iRBCs (37–39). In addition to reducing parasite burden (37–39) and inducing antibody and cell-mediated immune responses, the spleen is also responsible for phagocytosis and presentation of cleared parasites to resident CD4^+^ and CD8^+^ T cells (40) which then migrate to the brain and other organs *via* chemotaxis (41–43). Cytotoxic CD8^+^ T cells responsible for blood–brain barrier disruption were indeed shown to be primed in the spleen (44), in response to blood-stage infection and in an antigen-specific manner (37, 40, 45, 46), a factor that was crucial in ECM development (47).

Although most studies on ECM mainly focus on the intra-erythrocytic stages of infection, relatively little is known about the effects of pre-erythrocytic parasite development on the host immune response that contributes to the development of cerebral malaria. While the intra-erythrocytic stage of the parasite life cycle exclusively accounts for the severe pathology associated with *Plasmodium* infections, the pre-erythrocytic stage of the parasite life cycle is not associated with any of the symptoms of malaria. However, it has recently become evident that the immune response to malaria can vary dramatically according to the route of transmission, and the importance of replicating “natural transmission” is becoming clear (48, 49). During natural transmission, *Plasmodium* sporozoites are deposited in the skin through bites of infected mosquitoes. These sporozoites first go through an obligatory intra-hepatic phase before emerging into the blood stream, where they initiate the next round of replication within erythrocytes. Emerging evidence from rodent studies suggest that pre-erythrocytic or early immune responses may reduce the symptoms of ECM (50, 51) but the mechanisms by which this may occur are unclear. Moreover, recent studies from both rodents and humans have proposed that differences exist in the expression of surface antigens between blood-passaged and mosquito-passaged parasites (48, 52, 53) which also influences parasite virulence and the host immune response (48). Despite these differences, the effects of mosquito-passaged and blood-passaged parasites on the priming of antigen-specific CD8^+^ T cells in the spleen and subsequent development of ECM in infected animals had not been investigated.

In this study, we investigated an intra-hepatic and intra-erythrocytic stage antigen and describe its role in the development of ECM. By investigating contrasting infections initiated with either sporozoite or blood-stage parasites, we found that the contribution of antigen-specific responses to ECM development can vary between both modes of infection. We propose that the development of ECM differs for sporozoite and iRBC-induced infections and thus highlight limitations of studies focusing entirely on the intra-erythrocytic stage of the parasite life cycle.

## Materials and Methods

### Ethical Statement

All experimental animal procedures were performed in accordance with standard guidelines as set by regulations concerning FELASA category B and GV-SOLAS. Animal experiments were approved by the German authorities (Regierungspräsidium Karlsruhe, Germany), 1 8 Abs. 1 Tierschutzgesetz (TierSchG) under the license G-258/12. 6- to 8-week-old female C57BL/6J and NMRI mice were purchased from Janvier, France and kept under specific pathogen-free conditions at the animal facility (IBF) of the University of Heidelberg.

### Mice and Mosquito Infections

*Pb*ANKA and *PbmaLS_05* (−) parasites were maintained in mice and *Anopheles stephensi* mosquitoes by cyclical transmission. *Anopheles* mosquitoes were permitted to feed on infected mice after gametocyte exflagellation was observed in a drop of tail vein blood as previously described (54) and then maintained at 21°C and 80% humidity. Sporozoites were dissected from the salivary glands of infected female mosquitoes 17–21 days post-blood meal, as described previously (55) for *in vitro* and *in vivo* infections.

C57BL/6J mice were infected with *Pb*ANKA or *PbmaLS_05* (−) parasites through intravenous injections of 10^4^ salivary gland sporozoites or bites of 10 infected female mosquitoes. In separate experiments, mice were intravenously injected with 10^6^ iRBCs taken from sporozoite-infected donors. Parasitemia was monitored daily by Giemsa-stained blood smears and pre-patency determined by measuring the time from infection to first appearance of parasites in the blood. All mice were monitored for signs of ECM every day using the rapid murine coma and behavioral scale as previously described (56), and euthanized when signs of cerebral pathology were evident.

### Transcriptional Analysis of the *PbmaLS_05* Gene During Different Stages of Parasite Life Cycle

Samples were collected from sporozoites isolated from mid-guts and salivary glands of *Pb*ANKA infected mosquitoes at day 14 and 17 respectively, mixed blood stages from *Pb*ANKA infected mice, blood-stage schizonts from an overnight blood culture as previously described (57), and *in vitro* pre-erythrocytic stages harvested at 24, 48 and 63 hours post-infection (hpi). Pre-erythrocytic stages were obtained by infecting a monolayer of adherent HuH7 cells cultured in an 8-well labtek, with sporozoites isolated from salivary glands of *Pb*ANKA-infected mosquitoes. After 90 min of invasion time, the excess sporozoites were aspirated and the wells replenished with fresh DMEM culture medium supplemented with 10% FCS and 1% Antibiotic-Antimycotic (Gibco). The cells were harvested at 24, 48, and 63 hpi, lysed in TRIZOL, and stored at −80°C until RNA isolation. Total RNA was extracted using the RNAeasy kit (Qiagen), according to the manufacturer’s instructions. cDNA was synthesized from total RNA using a mixture of random and oligo dT primers and MMulV reverse transcriptase enzyme according to instructions provided in the First Strand cDNA synthesis kit (Thermo Scientific). A transcriptional analysis for different stages of the parasite life cycle was performed using primer pairs designed to cover the full-length gene (listed in Table S1 in Supplementary Material) and amplify fragments of 300–700 base pairs each, as shown in Figure S1C in Supplementary Material.

For quantification of parasite load in the brain, we sacrificed *Pb*ANKA WT or *PbmaLS_05* (−)-infected mice 8 days after sporozoite infection and 5 days after injection with iRBCs. The brains were harvested after perfusion with PBS and homogenized in 3 ml of TRIZOL. Quantification of parasite load in the brain was done by qRT-PCR for parasite-specific 18S rRNA transcripts and normalized to mouse GAPDH as previously shown (58) [primers are listed in Table S1 in Supplementary Material]. Relative copy numbers were determined *via* the ΔΔCT method (59).

### Generation of *PbmaLS_05* (−) Parasites

The *PbmaLS_05* gene locus was targeted by double homologous recombination. 500 bp fragments from the 5′ and 3′ UTR of the *PBANKA_140100* gene were amplified from *P. berghei* ANKA genomic DNA using primers listed in Table S1 in Supplementary Material and both fragments cloned into a targeting vector derived from the pBAT/pBART generation (60). The resulting plasmid was linearized with enzymes *Xho I* & *Sac II* and gel purified. Blood-stage schizonts purified from a density gradient were transfected with the linearized construct by electroporation using the Amaxa nucleofactor kit (Lonza), and then intravenously injected into mice (57). Transfected parasites were selected by addition of pyrimethamine to the drinking water and stable integration into the genome confirmed by primer pairs as depicted in the diagram (Figure 3A). A clonal population of *PbmaLS_05* (−) parasites were selected for *in vivo* by limiting dilution and injection of a single iRBC per mouse (61). Purity of the clonal population was assessed by the absence of a PCR product after amplification of the wild-type *PbmaLS_05* locus (Figures S3A,B in Supplementary Material). A similar strategy was used to generate *PbmaLS_05* (−) parasites in the *Pb*GFP Luc_con_ parasite strain (*P. berghei* line 676m1c11) (62), in order to visualize parasite distribution *in vivo via* whole body imaging of bioluminescence. Primer pairs used for PCR amplification and genotyping are listed in Table S1 in Supplementary Material.

### Generation of *PbmaLS_05* CT EGFP Parasites

The construct was designed to introduce an EGFP tag at the C terminal end of *Pb*maLS_05, using the double homologous crossover strategy. A 500 bp fragment corresponding to the 3′ end of the *PbmaLS_05* open reading frame (ORF) without the stop codon and a 523 bp fragment from the 3′ UTR region were amplified from *P. berghei* ANKA genomic DNA, using primers listed in Table S1 in Supplementary Material. The PCR fragment was then cloned into a b3D + EGFP vector upstream of the EGFP. The resulting plasmid was linearized by an over-night digest with enzymes *Hind III* and *Bam HI*, gel purified and transfected into purified blood-stage schizonts (57). The selection and cloning procedures were carried out as previously described (61). Stable integration was confirmed using primers that bound upstream of the integration site and downstream of the stop codon. Expression of EGFP was analyzed by fluorescence microscopy.

### Preparation and Microscopy of Pre-Erythrocytic and Blood-Stage Parasites

HuH7 cells were maintained at 37°C, 5% CO_2_ in DMEM culture medium supplemented with 10% FCS and 1% Antibiotic-Antimycotic (Gibco). To assess parasite growth at 24, 48, and 63 h post-infection, we fixed *in vitro* pre-erythrocytic stages at 24, 48, and 63 hpi, respectively with ice-cold methanol for 10 min, at room temperature. The cells were then blocked with 10% FCS in PBS and stained with *Plasmodium*-specific anti-HSP70 antibody (63), or rabbit anti-acyl carrier protein (ACP) antibody (64) and visualized by the addition of goat anti-mouse secondary antibody coupled to Alexa Fluor 488. After the addition of Hoechst, the cells were mounted in 30% glycerol, sealed with a coverslip and imaged on a fluorescence microscope (Zeiss). All images were processed using the ImageJ software (Version 2.0) for the addition of scale bars and measurement of parasite size.

Blood-stage schizonts were fixed with 4% PFA containing 0.0075% glutaraldehyde, then permeabilized with 125 mM glycine containing 0.1% Triton-X 100. After blocking with 3% BSA, the fixed schizonts were probed using anti-GFP (Abcam) and rabbit anti-ACP antibodies and visualized by the addition of appropriate secondary antibodies coupled to Alexa Fluor 488 against GFP and Alexa Fluor 546 against ACP. The nuclei were stained with Hoechst, the cells mounted in PBS and imaged on a spinning disk confocal microscope (Perkin Elmer ERS-VoX) under a 63× objective. The acquired images were processed using the ImageJ software.

Intra-hepatic stages of *Pb*maLS_05 CT EGFP parasites were visualized live at 24, 48, and 60 hpi under the 63× objective of a spinning disk confocal microscope (PerkinElmer ERS-VoX). The medium in the wells was replaced with fresh culture medium containing Mitotracker and Hoechst half an hour before imaging. Merosomes collected from *in vitro* intra-hepatic cultures as previously described (65) and blood-stage schizonts obtained from blood-cultures were also labeled with Mitotracker and Hoechst and mounted in PBS for live imaging in a pre-incubated chamber at 37°C with 5% CO_2_. Stacked images were recorded and later processed using the ImageJ software.

In order to disrupt branching of the apicoplast, intra-hepatic cultures were treated with 1 µM azithromycin [TEVA GmbH (58)]. The culture medium in the wells was refreshed every 24 hpi with medium containing the drug. Hoechst and Mitotracker were added to the wells, half an hour before imaging. To improve resolution, all images were deconvoluted using the AutoQuant X software and later processed using ImageJ.

### Bioluminescence Imaging

Whole body imaging was performed as previously described (66). For *in vivo* imaging of liver load, infected mice were briefly anesthetized with isoflurane and their bellies shaved. 2.5 mg/ml of d-Luciferin (Synchem Laborgemeinschaft OHG, Germany) was injected intraperitoneally into each mouse just prior to imaging (IVIS 100; Caliper Life Sciences). For *ex vivo* imaging of brains, all infected mice were sacrificed and perfused intra-cardially with PBS. Brains were isolated and incubated for 10 min in a falcon containing 4 ml of PBS with 200 µl luciferin. The brains were then placed in a petridish and imaged. Bioluminescence was acquired after 2 min exposure time with medium binning factor and FOV of 12.5 cm. All images were analyzed using the region of interest (ROI) tool of the Living Image software (V2.50.1, Xenogen, Hopkinton, MA, USA). The ROI is expressed as total flux [photons (p)/second (s)].

### Magnetic Resonance Imaging (MRI)

Magnetic resonance imaging was performed on a 9.4 T small animal scanner (BioSpec 94/20 USR, Bruker Biospin GbmH, Ettlingen, Germany) using a volume resonator for transmission and a 4-channel-phased-array surface receiver coil. Anesthesia was induced by inhalation of 2% isoflurane and then maintained at 1–1.5%. The mice were placed prone in a fixed position with continuous monitoring of body temperature and respiration. MRI scans were performed at the peak of neurological symptoms, which was at day 5 in blood-stage-infected mice (wildtype *n* = 6, mutant *n* = 7) and at day 7 in sporozoite-infected mice (wildtype *n* = 6, mutant *n* = 7). Additionally, control mice (*n* = 6) were also examined to determine baseline values. The MR imaging protocol included 3D T1-weighted (w) imaging (TR/TE = 5/1.9 ms, FA = 8.5°, and 156 µm isotropic resolution), T2*-w flow compensated gradient echo imaging (TR/TE = 50/18 ms, FA = 12°, and 80 µm isotropic resolution) and 2D T2-weighted imaging (TR/TE = 2,000/22 ms, slices = 12, and slice thickness = 0.7 mm). Image processing was undertaken in Amira 5.4 (FEI, Visualization Sciences Group). Brain volume was assessed semi-automatically on 3D T1-w datasets. Brain edema was quantified by signal ratio measurements. Microhemorrhages were analyzed on T2*w images. Grading of microhemorrhage load into mild (=1), moderate (=2), and severe (=3) was performed as previously published (34).

### Histology

Brains were carefully harvested after perfusion with PBS, fixed in 4% PFA and embedded into paraffin blocks for sectioning as previously described (34). After cooling the paraffin blocks at −20°C for approximately 30 min, brains were cut into 1 µm thick coronal sections at 4 levels using a microtome. In order to reduce wrinkles, the sections were stretched in a waterbath (45°C). After staining with conventional Giemsa, the tissue slides were examined on an Olympus BX45 research microscope (Olympus, Tokyo, Japan).

### Isolation of Splenocytes and Brain-Infiltrating Lymphocytes for Flow Cytometry

Naïve, *Pb*ANKA WT, and *PbmaLS_05* (−)-infected mice were sacrificed and perfused intra-cardially with 20 ml of 1× PBS. The spleens and brains were then harvested and prepared as single cell suspensions for flow cytometry analysis. The spleens were homogenized with a pestle in complete RPMI medium (containing 10% FBS, 1% penicillin-streptomycin, 1 mM sodium pyruvate, and 1× MEM NEAA, all from Gibco) and passed through a 70 µm mesh to obtain a single cell suspension. After centrifugation, the cell pellet was treated with RBC lysis buffer (8.26 g NH_4_Cl, 1 g KHCO_3_, and 0.037 g EDTA in 1 l ddH_2_O, pH 7.5), washed once and then resuspended in complete medium. The brains were incubated in RPMI containing 0.5 mg/ml collagenase and 10 µg/ml DNAse I for 45 min at room temperature, then homogenized with a pestle and passed through a 70 µm filter. The suspension was centrifuged at 500 rpm for 30 s and the supernatant overlaid on a 30% percol gradient. After centrifugation at 1,900 *g* × 10 min, the pelleted cells were treated with RBC lysis buffer, washed once and resuspended in complete medium. 100 µl of brain and spleen cell suspensions were added to duplicate wells of a 96-well plate and stimulated with 1 µM of Pb1 peptide of GAP50 (SQLLNAKYL) (45) in the presence of 10 µg/ml Brefeldin A, or incubated with 10 µg/ml Brefeldin A (BFA) alone. After an incubation time of 5 h at 37°C, the plate was centrifuged to spin down the cells, supernatant discarded, and the cells washed once with 100 µl PBS to remove any residual peptide or BFA. The cells were then re-suspended and stained with anti-CD8-PE Cy7 (clone 53-6.7, eBioscience) and anti-CD4-PerCP Cy 5.5 (clone R35-95, BD Pharmingen) antibodies in the presence of FCR-block in a total volume of 30 µl PBS, for 20 min on ice. Following the addition of 100 µl PBS, the cells were pelleted and permeabilized by re-suspension in 100 µl 2% PFA at RT for 15 min in the dark. In the next step the cells were washed once, and stained with anti-IFN-γ-APC-Cy7 (clone XMG1.2, BD Pharmingen) including FCR-block in saponin buffer (0.1% BSA, 0.3% saponin in PBS), for 20 min on ice. Afterward, the cells were washed once with 100 µl PBS, centrifuged and re-suspended in 100 µl PBS for flow cytometry. The data were acquired on a FACS Canto I flow cytometer (BD Biosciences) and analyzed using the FlowJo software (version 10).

### Cross-Presentation Assay

C57BL/6J mice were infected by intravenous injection of 10^4^ sporozoites or by intraperitoneal injection of fresh, diluted mouse blood containing 10^6^ iRBCs of either *Pb*ANKA WT or *PbmaLS_05* (−) parasites. All sporozoite-infected mice were sacrificed 8 d.p.i. while iRBC-infected mice were sacrificed 5 d.p.i. Naïve mice were also sacrificed along with each group and served as controls. The isolation of brain microvessels and quantification of cross-presentation of the parasite-derived Pb1 epitope using TCR-transduced lacZ reporter cells (45) was done according to the previously described protocol (67). Reporter cells that had been activated by Pb1-MHC complexes present on the brain microvessel fragments were visualized as blue spots after X-gal staining.

### ELISpot Assay

SYPEITHII^1^ and BIMAS^2^ algorithms were used to predict CD8 T cell epitopes for the full-length *Pb*maLS_05 protein sequence, in both H-2K^b^ and H-2D^b^ backgrounds. Predicted CD8 T cell epitopes with the highest score and the Pb1 epitope of GAP50 were synthesized as peptides by JPT Peptide Technologies GmbH (listed in Table S2 in Supplementary Material). The peptides were dissolved in DMSO to a final stock concentration of 20 mM, aliquoted, and stored at −80°C. For pulsing, splenocytes isolated from naïve donors were used as antigen-presenting cells (APCs) and incubated with a pool of maLS_05 peptides (final concentration 10 µM), or GAP50 (10 µM) or unpulsed (DMSO control), for 60 min at 37°C with intermittent shaking. After incubation, the APCs were washed, counted, and adjusted to 10^5^ per well before plating in an ELISpot plate (Millipore) pre-coated with anti IFN-γ antibody (eBioscience). 2 × 10^5^ effector cells isolated from the brains and spleens of naïve, *Pb*ANKA, and *PbmaLS_05* (−)-infected mice were subsequently added to each well and cultured for 22–24 h. Due to insufficient numbers of brain-infiltrating lymphocytes, the total number of cells isolated was divided between unpulsed and pulsed wells. Polyclonal stimulation with α-CD3 antibody or PMA/ionomycin was used as positive controls. Secreted IFN-γ was detected after incubation with biotinylated anti-IFN-γ antibody (eBioscience) and streptavidin-ALP (Mabtech). Spots were subsequently visualized by the addition of a substrate solution (AP Conjugate Substrate Kit, Biorad) as per the manufacturer’s instructions, and counted using an ELISpot reader. We defined spot-forming units above the respective naive control as positive values. Spot counts for brain-infiltrating lymphocytes were calculated to 2 × 10^5^ effector cells, in order to allow comparison between groups.

### *In Vivo* Cytotoxicity of *PbmaLS_05*-Specific CD8^+^ T Cells

C57BL/6J mice infected with *Pb*ANKA sporozoites were treated with 0.8 mg of chloroquine (CQ; chloroquine diphosphate salt; Sigma-Aldrich) 7 and 8 days post-infection (p.i.) and with 0.1 mg artesunate (ART; Sigma-Aldrich), 7 and 7.5 days post-infection, while *Pb*ANKA iRBC-infected mice were treated with CQ 5 and 6 days p.i. and with artesunate 5 and 5.5 days p.i. All mice were intravenously injected thrice (4 h apart) with 100 µg of a pool of *Pb*maLS_05 peptides (11.11 µg of each of the 9 peptides listed in Table S2 in Supplementary Material) or 100 µg of Pb1 peptide, 2 h after the last drug treatment. All mice were challenged 24 h after the last peptide injection with two injections of 5 mg/ml folic acid (50 mg/ml folic acid dissolved in PBS with pH adjusted to 7.2; Sigma-Aldrich), 1 h apart and observed for up to 90 min (68).

### Statistical Analyses

Statistical significance was determined with GraphPad Prism (GraphPad Software version 5, La Jolla, CA, USA). The Shapiro–Wilk test was used to check for normal distribution of the data, while the GraphPad outlier calculator was used to check for outliers. Statistical difference between two groups was calculated using a Student’s *t*-test (for normally distributed data) or the Mann–Whitney *U*-test (for data that was not normally distributed), while that for three or more groups calculated by means of nonparametric tests (Kruskal–Wallis followed by Dunn’s multiple comparison for data that was not normally distributed or One-way ANOVA followed by Bonferroni correction for normally distributed data). *p*-values <0.05 were considered statistically significant.

## Results

### maLS_05 Localizes to the Apicoplast of Blood- and Liver-Stage Schizonts

**Figure 1:**
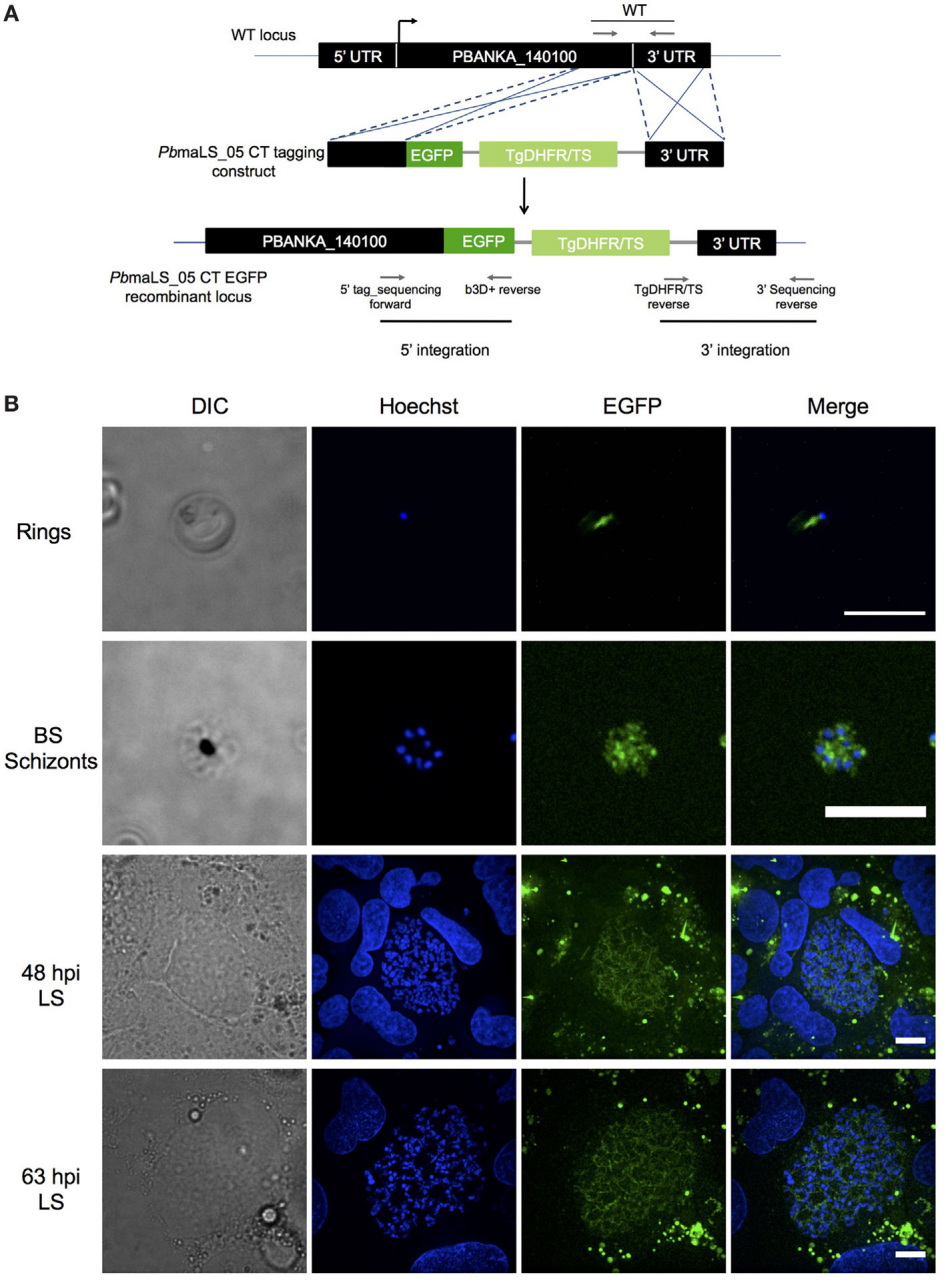
*Pb*maLS_05 is expressed in late liver- and blood-stage schizonts. **(A)** *Pb*maLS_05 EGFP parasites were generated by a double homologous crossover strategy that introduced a single EGFP tag at the C-terminal end of “ORF a.” **(B)** *Pb*maLS_05 is expressed in late blood- and liver-stages as observed by live imaging of *Pb*maLS_05 EGFP parasites under a spinning disk confocal microscope. The nuclei were stained with Hoechst. The images are representative of two independent experiments. Scale bar = 10 µm.

**Figure 2:**
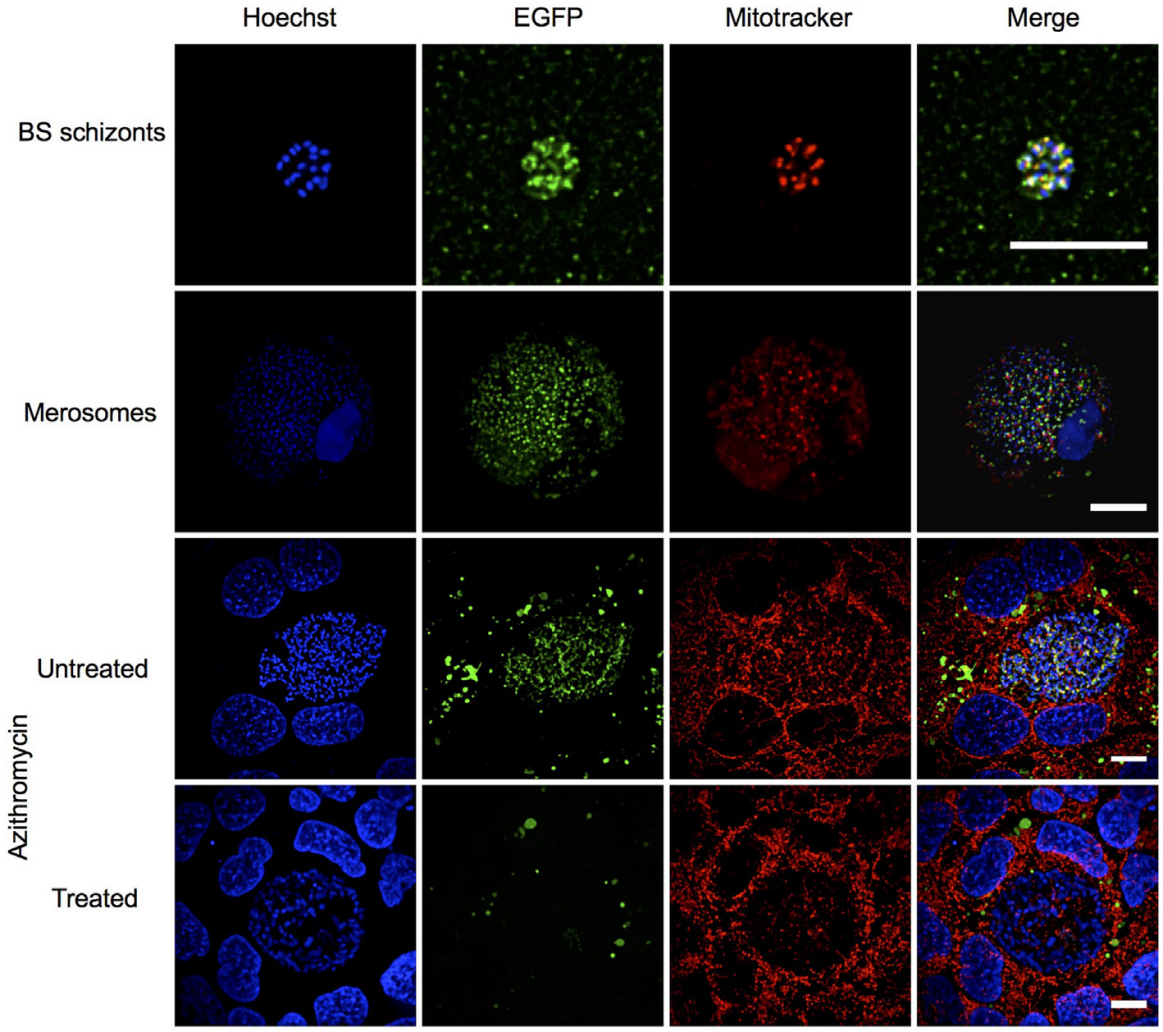
*Pb*maLS_05 localizes to the apicoplast of liver and blood-stage parasites. *Pb*maLS_05 EGFP parasites were co-stained with Mitotracker and imaged live using a spinning disk confocal microscope. *Pb*maLS_05 EGFP liver stages treated with 1 µM azithromycin did not show the branched structure for *Pb*maLS_05, as previously observed in untreated liver stage schizonts, thus confirming targeting of *Pb*maLS_05 to the apicoplast. The images displayed are representative of two independent experiments. Scale bar = 10 µm; for merosomes scale bar = 80 µm.

The ortholog of PBANKA_140100 (*Pb*maLS_05), i.e., PF3D7_1302500, was initially identified as a putative antigen that is differentially expressed in intra-hepatic stages of *P. falciparum* radiation-attenuated sporozoites in comparison to *Pf* wild-type (69). *PbmaLS_05* is well-conserved in all *Plasmodium* species on both genomic and proteomic levels (Figures S1A,B in Supplementary Material) and has a predicted protein size of 306 kDa that contains two predicted transmembrane domains and one predicted P loop containing nucleoside triphosphate hydrolase domain. In order to determine the expression profile of *Pb*maLS_05, we first examined the presence of *PbmaLS_05* transcripts in the different life-cycle stages of the parasite and found *PbmaLS_05* transcripts in all developmental stages (Figure S1C in Supplementary Material). To determine the localization of *Pb*maLS_05 in the parasite, we introduced a single C-terminal EGFP tag at the 3′ end of “ORF a” (Figure 1A). Clonal populations of transfected *Pb*maLS_05 EGFP parasites were isolated to determine expression and localization in the parasite life cycle (Figure S2A in Supplementary Material). Through live imaging of *Pb*maLS_05 EGFP parasites, we observed expression of the full-length *Pb*maLS_05 in rings, blood-stage- and liver-stage schizonts (Figure 1B). The localization was branched in intra-hepatic parasites during the cytomere stage of development and distinctly more punctuate in blood-stage schizonts, similar to what has previously been observed for parasite mitochondria and apicoplast (70) (Figure 1B). In comparison, no background fluorescence was detected for *Pb*ANKA WT liver stages (Figure S2B in Supplementary Material). Co-staining of *Pb*maLS_05 EGFP liver stage schizonts, merosomes and blood-stage schizonts with a mitochondrial marker, showed partial co-localization of the *Pb*maLS_05 protein with the mitochondria (Figure 2). However, treatment of *Pb*maLS_05 EGFP liver stages with azithromycin, a drug that inhibits biogenesis of the apicoplast (58, 70, 71) abolished the branched structure observed for *Pb*maLS_05, even though the mitochondrial structure was retained, suggesting that *Pb*maLS_05 localizes to the parasite apicoplast (Figure 2). To confirm that *Pb*maLS_05 localized to the apicoplast of blood-stage parasites, we co-stained *Pb*maLS_05 EGFP blood-stage schizonts with antibodies against GFP and ACP, an apicoplast-specific protein (72). Co-localization of both antibodies as observed by the yellow signal in *Pb*maLS_05 EGFP but not *Pb*ANKA WT schizonts confirmed that *Pb*maLS_05 indeed localizes to the apicoplast of blood-stage schizonts (Figure S2C in Supplementary Material).

### Deletion of *Pb*maLS_05 Has no Influence on Intra-Hepatic Development Both *In Vitro* and *In Vivo*

**Figure 3:**
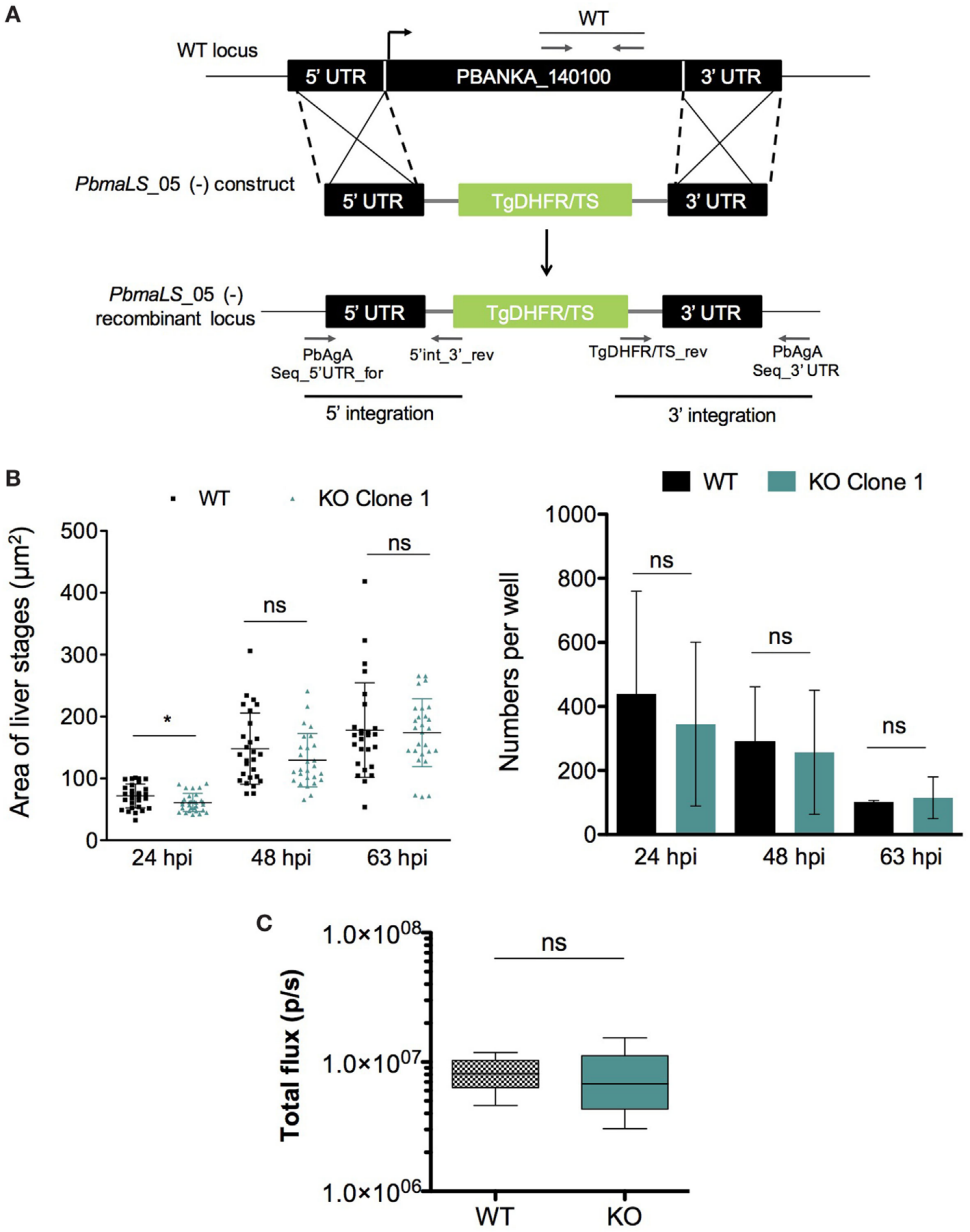
Deletion of endogenous *PbmaLS_05* does not impair intra-hepatic development both *in vitro* and *in vivo*. **(A)** The endogenous *Pb*maLS_05 locus was targeted by homologous recombination with a linearized plasmid consisting of 500 bp fragments of the 5′ and 3′ UTR, flanking a *Toxoplasma gondii* DHFR/TS cassette. **(B)** Intra-hepatic development is comparable between WT and KO parasites *in vitro*. HuH7 cells infected with 10^4^ sporozoites of either WT or KO parasites were fixed, 24, 48, and 63 h post-infection, respectively and stained with parasite-specific α-HSP70 (green) and Hoechst (blue) to visualize and quantify the intra-hepatic stages, by fluorescence microscopy. Images of intra-hepatic parasites at different time points post-invasion were recorded and sizes of liver stages measured using ImageJ. Mean ± SD values shown in the graph represent pooled values from two independent experiments. Statistical significance was determined using the unpaired student’s *t*-test with Welch’s correction, *p < 0.01. **(C)** No difference in parasite load in the liver between WT and KO parasites *in vivo*. 5 C57BL/6J mice were intravenously infected with 10^4^ sporozoites of *Pb*GFP Luc_con_ WT or *PbmaLS_05* (*−*) GFP Luc_con_ parasites. Two days post-infection all mice were anesthetized and injected with luciferin before imaging using the IVIS imaging system. Bioluminescence was measured in total flux (p/s). The data shown are pooled from two individual experiments for the sporozoite infection (*n* = 9 mice per group) and one for the infected red blood cell infection (*n* = 5 mice per group), for which the statistical significance was determined using the Mann–Whitney *U*-test.

In order to determine the role of *Pb*maLS_05 in the parasite life cycle, we used a double homologous crossover strategy to replace the full-length *PbmaLS_05* with a selectable marker (Figure 3A). *PbmaLS_05*-deficient (−) (KO) parasite clones were obtained from two independent transfections (Figure S3A in Supplementary Material) and absence of any residual WT parasites confirmed by RT-PCR amplification of the different ORF fragments (Figure S3B in Supplementary Material). Viable clones of *PbmaLS_05* (−) parasites were easily obtained thus suggesting that *Pb*maLS_05 is dispensable for development during the blood-stage.

Since *Pb*maLS_05 was expressed in mid-late stages of intra-hepatic parasites, we investigated the effects of *Pb*maLS_05 deletion on intra-hepatic development. Quantification of both numbers and development through size measurements of *in vitro* exo-erythrocytic forms, suggested that deletion of *Pb*maLS_05 had no effect on the number or developmental size of intra-hepatic parasites, *in vitro* (Figure 3B). A minor developmental delay was observed for KO parasites at 24 h post-invasion but not after 48 and 63 h.p.i. (Figure 3B). Moreover, the branching of the apicoplast and segregation between daughter nuclei was also comparable between WT and KO intra-hepatic parasites, thus excluding any defect in apicoplast inheritance (Figure S4 in Supplementary Material).

We then evaluated parasite burden in the liver *in vivo*, by intravenous injections of mice with sporozoites of either wild-type *Pb*GFP Luc_con_ (WT) or *PbmaLS_05* (−) parasites (KO) generated in the wild-type *Pb*GFP Luc_con_ strain [henceforth termed *PbmaLS_05* (−) GFP Luc_con_]. Bioluminescence imaging at 48 h post-injection showed no difference in the total flux [expressed as photons (p)/second (s)] in the liver between WT- and KO-infected mice (Mean ± SD; WT, 8.277 × 10^6^ ± 2.579 × 10^6^; KO, 7.537 × 10^6^ ± 4.625 × 10^6^) (Figure 3C). In a separate experiment, we inoculated mice with WT or KO sporozoites through different infection routes such as natural transmission *via* mosquito bites, subcutaneous, or intravenous injections. In agreement with our earlier observations, all KO-infected mice became patent with blood-stage infection on the same day as those infected with WT sporozoites, regardless of the route of infection used (Table 1), indicating that deletion of *Pb*maLS_05 has no effect on both infectivity and intra-hepatic development. Similar outcomes were also obtained with an independent clone of KO parasites (Table 1). Based on these results, we excluded a role for *Pb*maLS_05 in intra-hepatic development, both *in vitro* and *in vivo*.

**Table 1:** Mice infected through various routes with *PbmaLS_05* (−) sporozoites show the same pre-patency as those infected with *Pb*ANKA WT.

Route of infection	WT pre-patency (days)	KO clone 1 pre-patency (days)	KO clone 2 pre-patency (days)
Intravenous	3–4 (*n* = 17)	3–4 (*n* = 15)	3–4 (*n* = 6)
Subcutaneous	4–5 (*n* = 14)	5 (*n* = 7)	4–5 (*n* = 7)
Natural transmission/mosquito bite	3 (*n* = 14)	3 (*n* = 7)	3 (*n* = 7)

*Groups of 5 C57BL/6J mice were infected with 10^4^ WT or KO sporozoites intravenously, subcutaneously, or through the natural route of transmission, i.e., infectious bites of 10 mosquitoes. The mice were monitored for blood-stage parasites from 3 days on post-infection (d.p.i.) by microscopic examination of blood smears and pre-patency calculated as the number of days until the mice become blood-stage positive. Experiments performed with KO clones from an independent transfection showed the same result (*n* = the number of mice)*.

### *PbmaLS_05* (−)-Infected Mice do not Develop ECM

**Figure 4:**
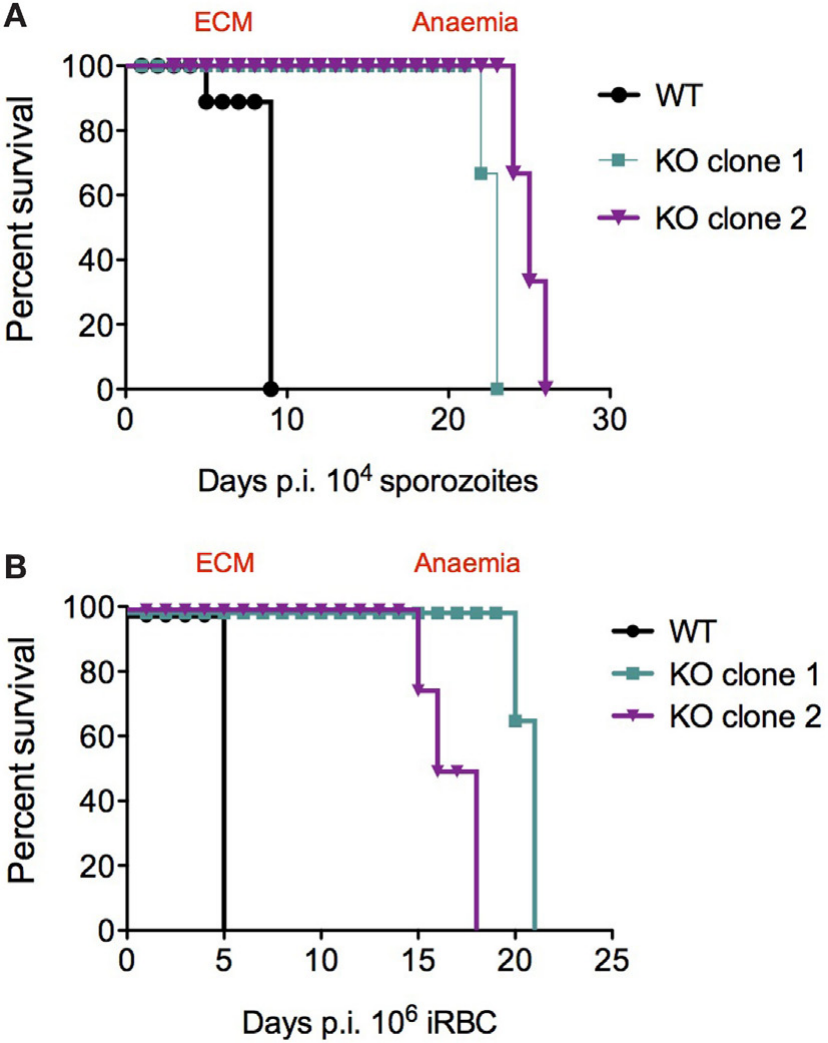
Survival plots for *Pb*ANKA WT and *PbmaLS_05* (−)-infected mice after sporozoite and infected red blood cell (iRBC) infections. KO-infected mice survive longer than WT-infected mice and do not develop ECM, after both sporozoite **(A)** and iRBC **(B)** infections. Similar results were observed for independent clones of KO parasites (see Table 2).

C57BL/6J mice infected with *Pb*ANKA sporozoites develop cerebral symptoms and succumb to ECM between day 7 and 10 post-infection. Interestingly, none of the mice infected with KO sporozoites developed ECM, but instead succumbed to hyperparasitemia-induced anemia on day 23–24 post-infection (Figure 4A; Table 2). In contrast, mice infected with WT sporozoites, displayed signs of ECM between day 7 and 9 p.i. (Figure 4A; Table 2). To examine if an impaired release of merosomes was responsible for protection from ECM (50, 51, 58), we bypassed the pre-erythrocytic stage and infected recipient mice with iRBCs taken from sporozoite-injected donors. Strikingly, none of the mice infected with KO iRBCs showed ECM symptoms, while the WT-infected mice succumbed to ECM around day 5 p.i. (Figure 4B; Table 3).

**Table 2:** Mice infected with *PbmaLS_05* (−) sporozoites do not develop experimental cerebral malaria (ECM).

Route of infection	WT		KO clone 1		KO clone 2	
	No. of mice	ECM (%)	No. of mice	ECM (%)	No. of mice	ECM (%)
Intravenous	17	100	15	0	8	0
Natural transmission/mosquito bite	14	85.58	7	0	7	0
Subcutaneous	14	78.57	7	0	7	0

*C57BL/6J mice infected with KO sporozoites failed to develop cerebral symptoms of ECM unlike mice infected with WT sporozoites, which succumbed to ECM between day 7 and day 10 post-infection. This phenotype was consistent regardless of the route of infection used*.

**Table 3:** *PbmaLS_05* (−)-infected mice do not develop experimental cerebral malaria (ECM) even after injection of iRBCs.

Group	Intravenous injection	Number of mice	ECM	% survival
WT	iRBC	18	17	11.11
KO clone 1	iRBC	11	0	100
KO clone 2	iRBC	6	0	100

*C57BL/6J mice were intravenously injected with 10^6^ infected red blood cells (iRBC) of either WT or KO parasites. Consistent with the phenotype observed after sporozoite injection, all mice injected with KO iRBCs did not develop ECM unlike WT-infected mice*.

**Figure 5:**
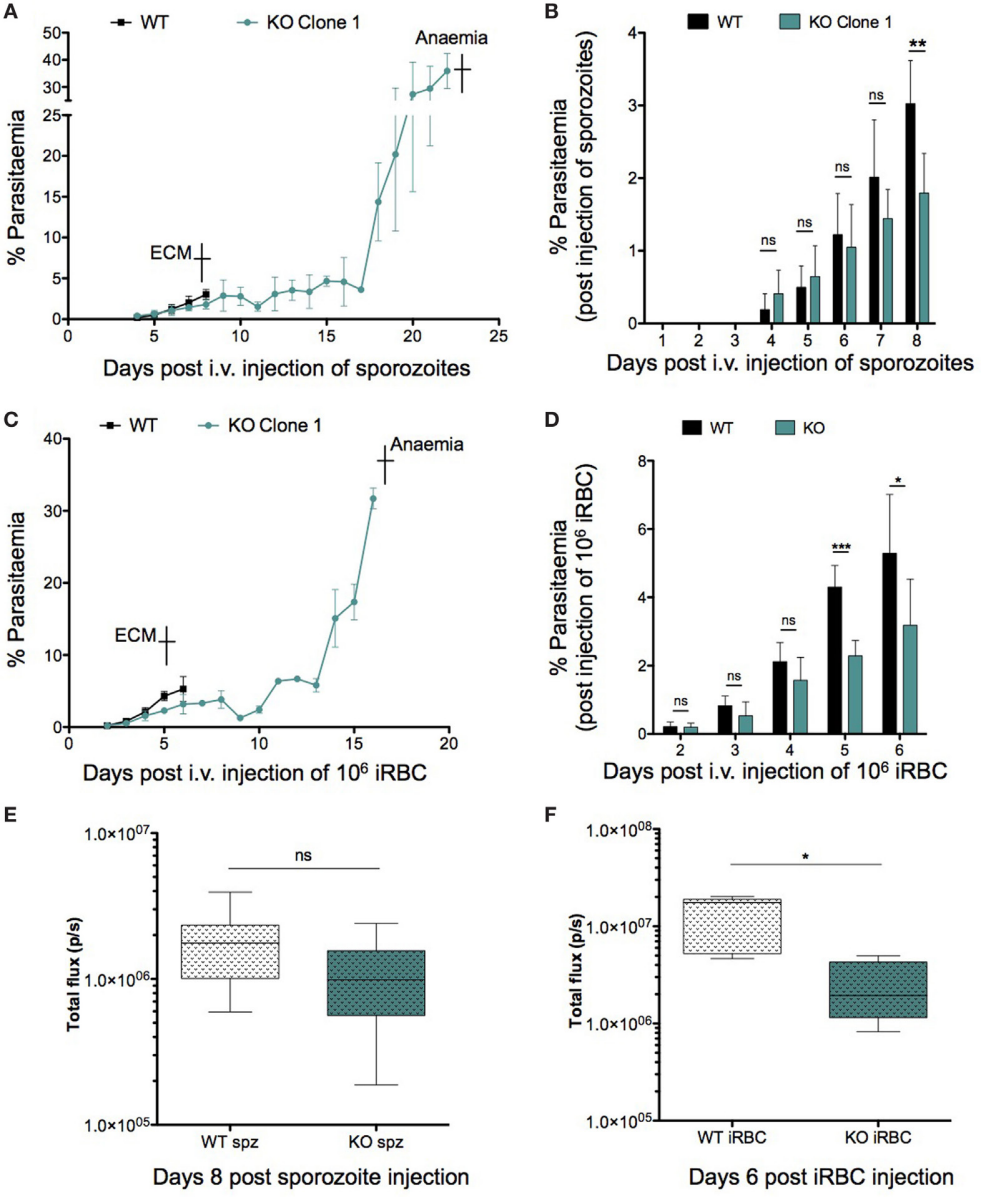
Role of parasitemia in experimental cerebral malaria (ECM) development. Groups of 3–5 C57BL/6J mice were infected with 10^4^ sporozoites or 10^6^-infected red blood cells (iRBCs) of WT or KO parasites, respectively. Parasite development in the blood was monitored from the day of patency until the endpoint, which was ECM for WT-infected mice and anemia for KO-infected mice. The data shown represents pooled values (Mean ± SD) from two independent experiments (*n* = 8 mice per group). **(A)** C57BL/6J mice infected with KO parasites succumbed to anemia caused by hyper-parasitemia in contrast to WT-infected mice, which died from ECM around day 8–9 p.i. of sporozoites. **(B)** Despite no difference in patent parasitemia (day 4 p.i.) the parasitemia in KO-infected mice was significantly lower compared to WT-infected mice, especially at the point when WT-infected mice developed ECM. **(C)** C57BL/6J mice were infected with 10^6^ WT or KO iRBCs and monitored for neurological signs. In contrast to WT mice which developed ECM and died around day 5 p.i., all KO-infected mice eventually died from hyper-parasitemia associated anemia between day 19 and day 22 p.i. **(D)** Parasite growth in the blood differs between WT- and KO-infected mice and is lower in KO-infected mice 5 and 6 d.p.i. when WT mice display signs of ECM. The Mann–Whitney *U*-test was used to determine statistical significance (**p < 0.001 and *p < 0.01; ns, not significant). **(E)** WT- and KO-infected mice show comparable parasite sequestration in the brain after sporozoite infection. 5 C57BL/6J mice were infected with 10^4^ *Pb*GFP Luc_con_ WT or *PbmaLS_05* (−) GFP Luc_con_ sporozoites. All WT-infected mice were sacrificed on day 8 p.i. when ECM symptoms were observed. After perfusion, the brains were isolated, incubated in PBS containing luciferin and imaged using the IVIS imaging system. Bioluminescence was recorded as total flux [photons (p)/second (s)]. The data are pooled values from two independent experiments (*n* = 9 mice per group). An unpaired Student’s *t*-test was used to determine statistical significance (ns, not significant). **(F)** Parasite sequestration in the brain is significantly lower for KO iRBC-infected mice when compared to mice infected with WT iRBC. 5 C57BL/6J mice were infected with 10^6^ iRBC of *Pb*GFP Luc_con_ WT or *PbmaLS_05* (−) GFP Luc_con_ parasites. All iRBC-infected mice were sacrificed on day 6 p.i. when WT-infected mice developed ECM. The brains were isolated after perfusion, incubated in PBS containing luciferin, and imaged using the IVIS imaging system. Bioluminescence was recorded as total flux [photons (p)/second (s)]. The data shown are from a single experiment (*n* = 5 mice per group). An unpaired Student’s *t*-test with Welch’s correction was used to determine statistical significance (*p < 0.01).

Since ECM development is associated with parasite load within organs such as the brain and spleen in addition to an intact host immune response, we decided to investigate both parameters to determine the reason behind the abrogation of ECM in KO-infected mice. We looked at peripheral blood parasitemia of mice infected with both WT and KO sporozoites and iRBCs. WT and KO sporozoite-infected mice showed no difference in pre-patency or significant difference in parasitemia during the first 6 days of infection (Figure 5A). Bioluminescence imaging at 72 h.p.i. confirmed a similar parasitemia between WT and KO sporozoite-infected mice [Mean ± SD; WT, 7.694 × 10^6^ ± 4.253 × 10^6^ photons (p)/second (s); KO, 8.409 × 10^6^ ± 3.5 × 10^6^] (Figure S5A in Supplementary Material). However, when WT mice showed signs of ECM 8 days after infection, the parasitemia in the KO sporozoite-infected mice was significantly lower than the WT-infected group (Figure 5B). Similar results were also observed with an independent clone of *PbmaLS_05* (−) parasites (Figures S5B,C in Supplementary Material). We then infected mice with 10^6^ WT or KO iRBC and recorded the parasitemia 2 days after infection until the point when KO-infected mice succumbed to anemia (Figure 5C). Despite no difference on day 2 p.i., all KO iRBC-infected mice had significantly lower parasitemias on day 5 and 6 p.i., at the time when WT mice developed ECM, similar to what we observed after sporozoite infection (Figure 5D).

We then looked for differences in parasite sequestration within the brains of WT and KO-infected mice after both sporozoite and iRBC injections. Bioluminescence imaging of the brains of WT and KO iRBC-infected mice at the onset of cerebral symptoms showed a significant reduction in parasite sequestration in the brain for the KO-iRBC-infected group (Figure 5F). This difference was not apparent between WT and KO-sporozoite-infected mice (Figure 5E), despite a reduction in peripheral parasitemia (Figure 5B). The results of the bioluminescence imaging were also verified by qRT-PCR analysis of brains isolated from WT- and KO-infected mice after both sporozoite and iRBC infections. Both WT- and KO-infected groups were sacrificed on the days when WT-infected mice displayed neurological signs and their organs harvested after perfusion. In agreement with the bioluminescence data, the reduction in parasite load in the brain was only observed for mice infected with KO iRBCs (Figure S5E in Supplementary Material). In contrast, the brains of both WT- and sporozoite-infected mice had similar parasite loads (Figure S5D in Supplementary Material).

**Figure 6:**
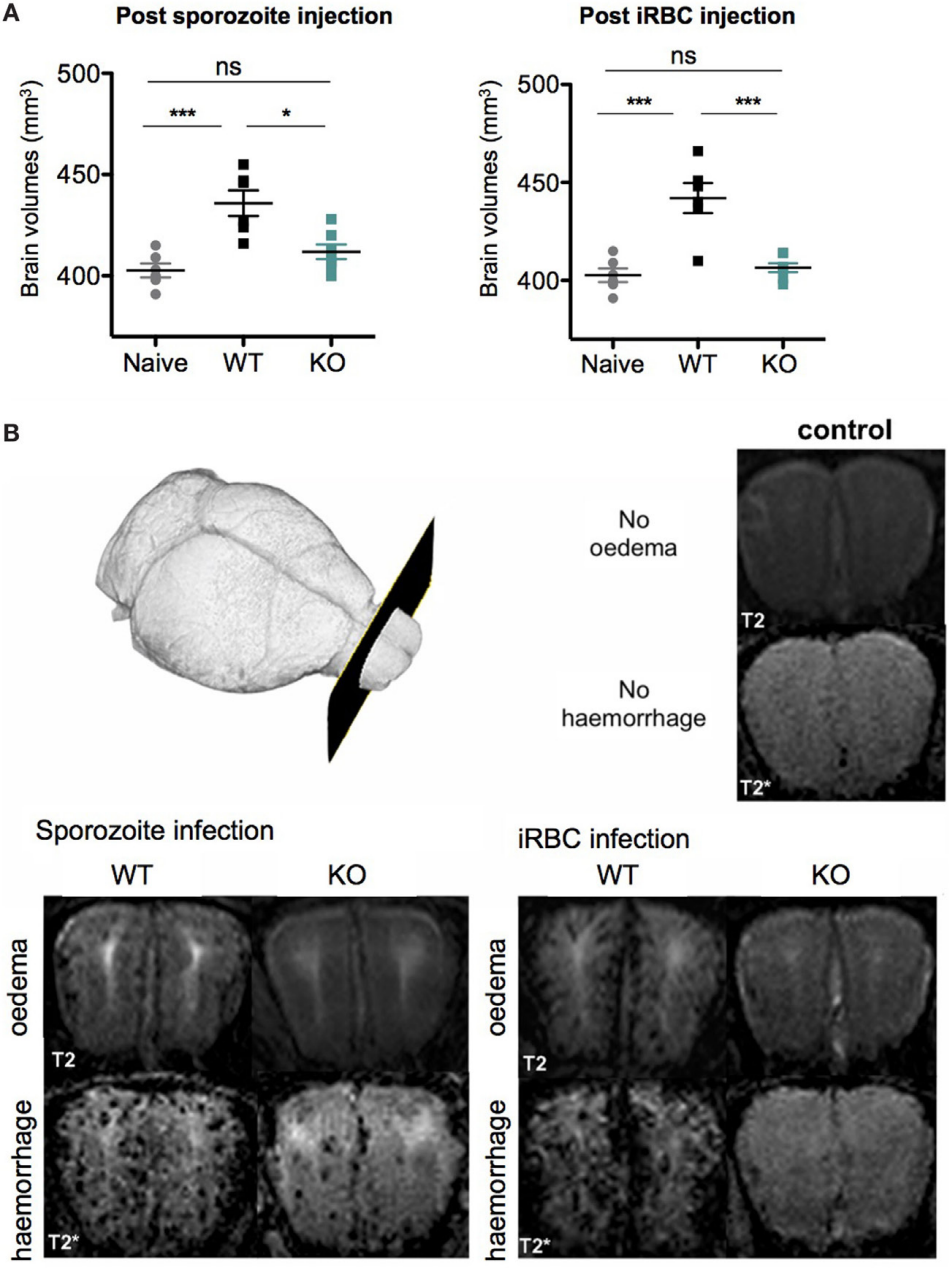
*PbmaLS_05* (−)-infected mice show mild-cerebral changes in the olfactory bulb on *in vivo* magnetic resonance images. **(A)** Brain volume in *Pb*ANKA WT mice with experimental cerebral malaria (ECM) symptoms is significantly increased compared to healthy control mice and KO-infected mice. Brain volumes of KO sporozoite-infected mice are slightly, but not significantly increased compared to healthy controls. The brain volumes represented as mean ± SEM are pooled values from two independent experiments. Statistical significance was determined using a One-way ANOVA with Bonferroni multiple comparison as a *post hoc* test (***p < 0.0001, **p < 0.001, and *p < 0.01; ns, not significant). **(B)** Representative images at the level of the olfactory bulb are shown. In healthy controls, no edema is seen on T2-weighted images nor are microhemorrhages seen on T2*-weighted images. In WT-infected mice both severe edema and a high microhemorrhage load are evident, while KO-infected mice display only mild changes in the olfactory bulb not leading to ECM. Comparing both KO groups less edema and less microhemorrhages are seen in KO-infected red blood cell-infected mice compared to KO sporozoite-infected mice.

To further examine differences in cerebral disease *in vivo* we analyzed cerebral brain swelling, edema, and microhemorrhage load by MRI. As expected, the MR images showed that ECM symptoms were pronounced in WT-infected mice after both sporozoite and iRBC infections. There was a significant increase in brain volume as compared to healthy controls (*p* = 0.001) for both groups of WT-infected mice (Figure 6A), which was consistent with a severe increase in edema, as evidenced by the signal to noise ratio (WT iRBC edema signal ratio 1.9 ± 0.09; WT sporozoites edema signal ratio 1.8 ± 0.1, *p* = 0.55). We additionally observed a severe microhemorrhage load in both WT iRBC and WT sporozoite-infected groups (WT iRBC score 2.8 ± 0.4; WT sporozoites score 2.6 ± 0.5, *p* = 0.55), consistent with what has been shown for mice with ECM (34). In contrast, mice infected with KO sporozoites and iRBC, which did not show ECM symptoms, displayed no significant increase in brain volume when compared to healthy control mice (Figure 6A). However, mild edema was observed in the olfactory bulb, a predilection site of ECM (34, 73), for both groups of KO-infected mice, although it was less pronounced in KO iRBC compared to KO sporozoite-infected mice (KO iRBC edema signal ratio 1.18 ± 0.05; KO sporozoites edema signal ratio 1.4 ± 0.2, *p* = 0.02). Furthermore, only few microhemorrhages were observed in both KO groups with significantly less microhemorrages in KO iRBC group compared to the KO sporozoite-infected mice (KO iRBC score 0.5 ± 0.4; KO sporozoites score 1.2 ± 0.5, *p* = 0.02) (Figure 6B). A similar degree of microhemorrhages in all groups was detected on histological brain tissue sections (Figure S6 in Supplementary Material). In summary, KO-infected mice showed mild cerebral pathology not mounting to ECM, which was more pronounced in the sporozoite-infected group compared to those infected with iRBC. The results of these experiments suggested that deletion of *Pb*maLS_05 has an effect on peripheral blood parasitemia after both sporozoite and iRBC infections, but alters parasite sequestration in the brain only after iRBC infection.

### *PbmaLS_05* (−) Sporozoite-Infected Mice do Not Develop ECM Despite Comparable Levels of Brain-Infiltrating Lymphocytes to Mice Infected With *Pb*ANKA Sporozoites

**Figure 7:**
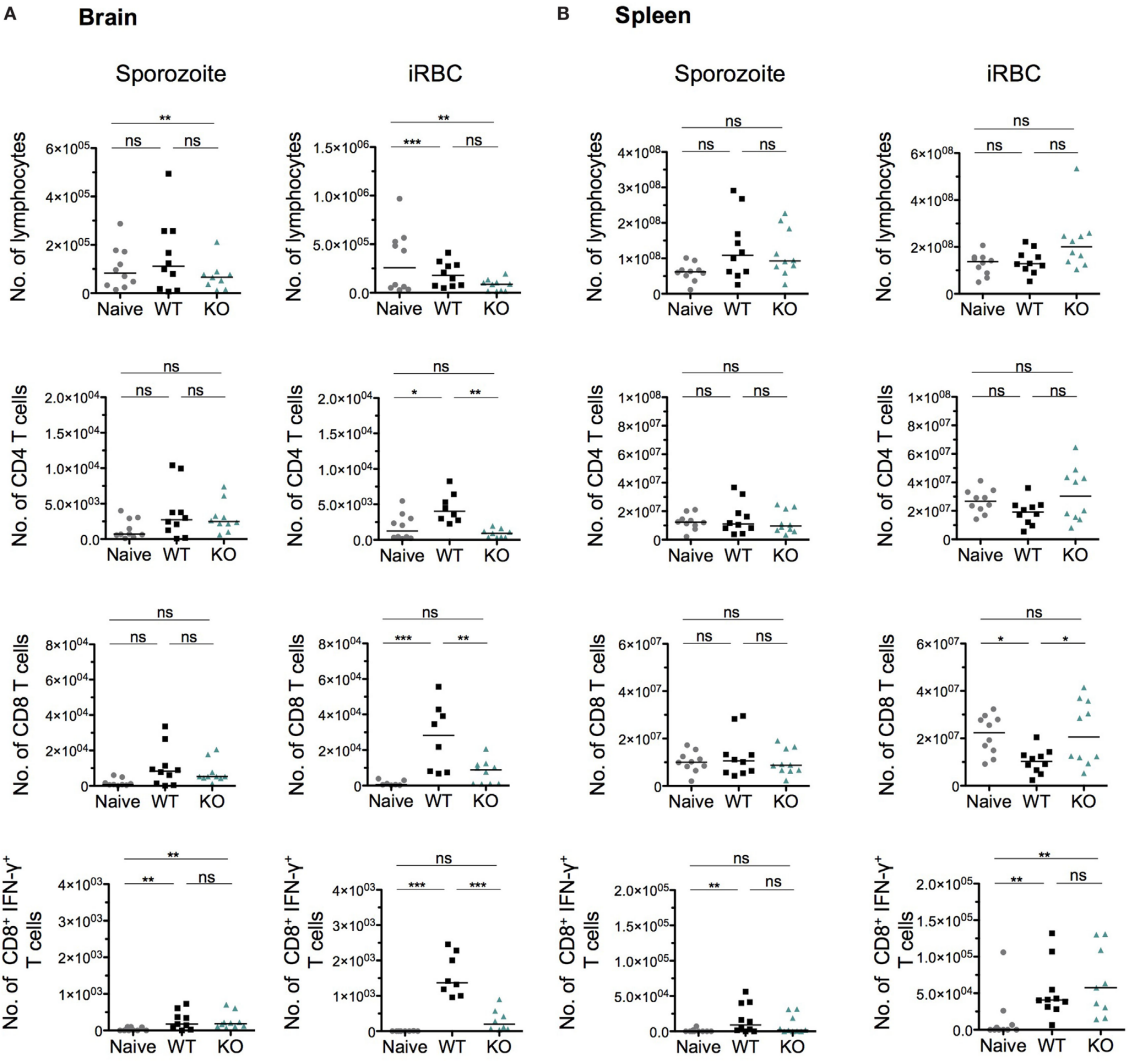
The immune status in spleen and brain is indistinguishable between *Pb*ANKA WT and *PbmaLS_05* (−)-infected mice after sporozoite injection, but significantly differs after infected red blood cell (iRBC) infection. Groups of five mice were infected with either WT or KO sporozoites and sacrificed on day 8 p.i along with five naïve mice. A separate set of mice were infected with WT or KO iRBCs and sacrificed on day 5 p.i. along with five naïve mice. Lymphocytes isolated from brains **(A)** and spleens **(B)** were stimulated *ex vivo* with Pb1 and stained with CD8, CD4, and IFN-γ antibodies for quantification and phenotypic analysis by flow cytometry. Each data point represents cell numbers isolated from one individual mouse and the results shown with median are pooled data from two independent experiments. Statistical significance was determined by Kruskal–Wallis test with Dunn’s multiple correction or One-way ANOVA with Bonferonni’s adjustment (***p < 0.0001, **p < 0.001, and *p < 0.01; ns, not significant).

To further characterize the absence of ECM after infection with KO sporozoites, we examined the host immune response in brains and spleens of WT- and KO-infected mice, after both sporozoite and iRBC injections. Several studies have implicated CD8^+^ T cells and to a lesser extent CD4^+^ T cells in blood–brain barrier disruption and pathogenesis of ECM (44, 74–77). Since parasite sequestration seemed unaffected in the brains of KO sporozoite-infected mice (Figure 5E), we hypothesized that an impaired T cell response in these mice might explain the ablation of ECM, possibly due to altered activation/priming of T cells in the spleen. We, therefore, quantified total CD4^+^ and CD8^+^ T cell numbers isolated from spleens and brains of mice infected with WT or KO sporozoites, 8 days p.i. To obtain one measure of the antigen-specific functional CD8^+^ T cell response, we also quantified the number of CD8^+^ T cells that were positive for IFN-γ produced in response to *ex vivo* stimulation with Pb1 peptide, a well-characterized highly immunogenic CD8^+^ T cell epitope present in the parasite protein GAP50 (45). Interestingly, both the total number of CD4^+^ and CD8^+^ T cells isolated from the brains and spleens of WT- and KO-sporozoite-infected mice were comparable (Figures 7A,B). In addition, no difference in the Pb1-specific T cell response was observed, suggesting that the antigen-specific response was not altered in KO-infected mice after sporozoite infection (Figures 7A,B). We then quantified T cells and measured Pb1-specific CD8^+^ T cell responses in brains and spleens of mice post-injection with iRBCs. In contrast to observations made after sporozoite inoculation, we found a significant reduction in the number of infiltrated CD4^+^ and CD8^+^ T cells in the brains of KO-iRBC-infected mice, along with reduced numbers of Pb1-specific IFN-γ^+^ CD8^+^ T cells when compared to mice infected with WT-iRBC (Figure 7A). The numbers of CD8^+^ T cells were higher in the spleens of KO-iRBC-infected mice, which corresponded to their reduced accumulation in the brain (Figure 7B). Although these results were in agreement with previous observations about parasite sequestration and supported the abrogation of ECM in KO-iRBC-infected mice, they did not offer any explanation for the absence of ECM in KO-sporozoite-infected mice.

### The Cross-Presentation of Pb1 by the Brain Endothelium in *PbmaLS_05* (−) Infected Mice Is Less Efficient

**Figure 8:**
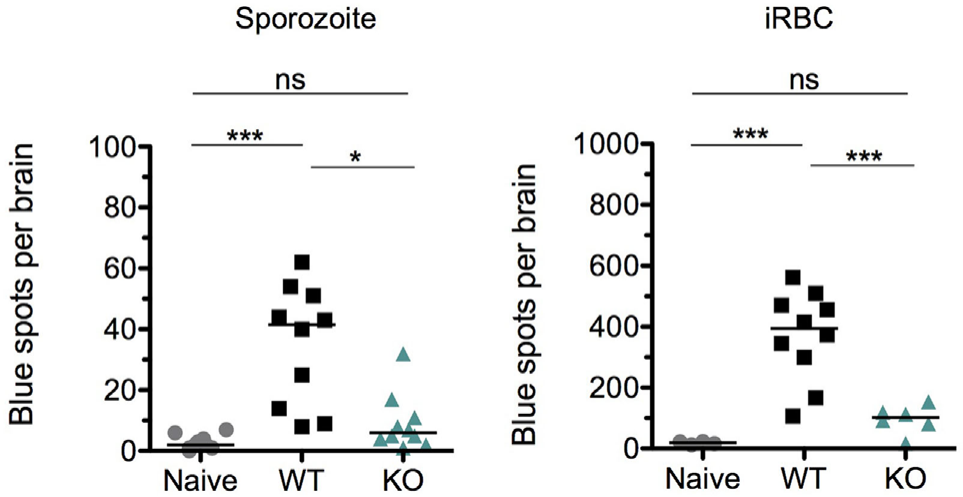
The cross-presentation of Pb1 by brain endothelial cells is reduced in KO-infected mice more after infected red blood cell (iRBC) than after sporozoite infection. Groups of 5 C57BL/6J mice were infected with 10^4^ sporozoites or 10^6^ iRBC of WT or KO and harvested when WT-infected mice exhibited neurological signs, i.e., on day 8 post-sporozoite injection and day 6 post-iRBC injection. An additional group of naïve mice were harvested for the assay. Brain microvessel fragments were isolated and co-incubated with reporter cells that express lacZ upon detection of a parasite-derived peptide-MHC complex (*Pb*GAP50; SQLLNAKYL). Blue spots representing activated reporter cells were quantified following X-gal staining. The data shown are pooled values from two independent experiments with each point representing individual mice. The bars represent medians. One-way ANOVA was used after sporozoite infection while Kruskal–Wallis was used after iRBC infection to determine statistical significance; ***p < 0.0001, **p < 0.001, and *p < 0.01.

Aside from an intact host immune response, previous studies have shown that the cross-presentation of parasite antigens by the activated brain endothelium is a critical factor that distinguishes ECM from non-ECM causing parasite strains (45, 78). Because parasite sequestration and the numbers of immune effector cells were comparable between WT- and KO-sporozoite-infected mice (Figures 7A,B), we hypothesized that perhaps altered cross-presentation of parasite antigens by the activated brain endothelium could account for the abrogation of ECM in KO-sporozoite-infected mice. Since the cross-presentation of the Pb1 epitope of GAP50 was shown to play an important role in the induction of ECM (45) and due to the availability of the Pb1 reporter cell line, we decided to measure the cross-presentation of Pb1 by brain microvessels isolated from WT and KO sporozoite-infected mice. For comparison, we also performed the cross-presentation assay with microvessels isolated from mice after iRBC infection. In good agreement with the immunological data and reduced parasite numbers post-iRBC infection, the level of cross-presentation of the Pb1 epitope by the brain endothelium was indeed significantly lower in the KO iRBC-infected mice (Figure 8). Interestingly, the level of cross-presentation of Pb1 by microvessels isolated from KO-sporozoite-infected mice was lower when compared to that of WT-infected mice (Figure 8), though not as significant as after iRBC infection. This was indeed surprising given that we did not observe any difference in parasite sequestration (Figure 5E) or infiltration of Pb1-specific IFN-γ^+^ CD8^+^ T cells in the brain after sporozoite infection (Figure 7A).

Thus, these data suggested that the cross-presentation of parasite antigens by endothelial cells in the brain is altered in KO-infected mice, after both sporozoite and iRBC infections.

### *Pb*maLS_05-Specific CD8^+^ T Cells Contribute to ECM Development After a Sporozoite Infection

**Figure 9:**
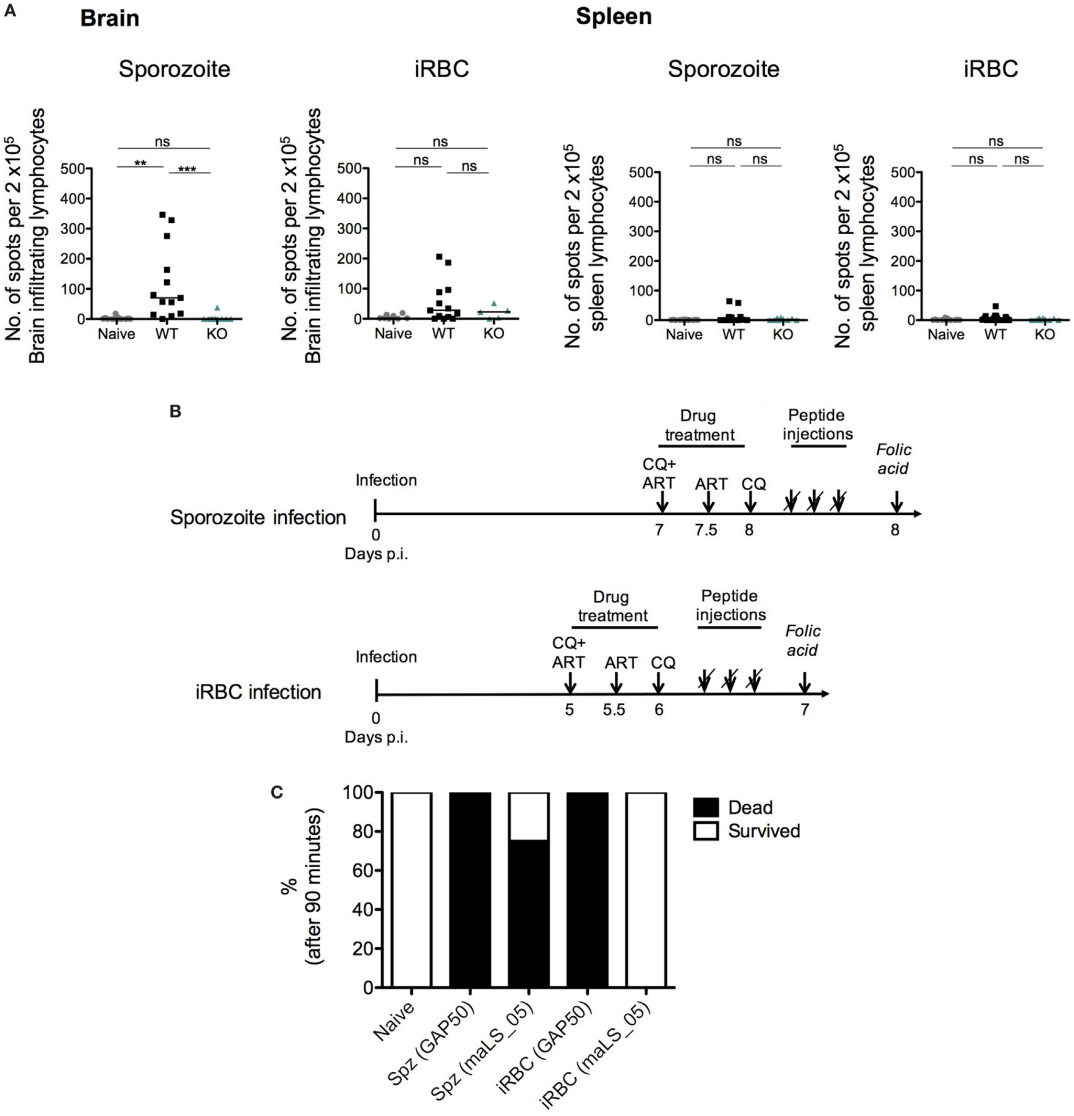
*Pb*maLS_05-specific T cells contribute to the development of experimental cerebral malaria (ECM), after sporozoite- but not infected red blood cell (iRBC)-induced infection. **(A)** *Pb*maLS_05-specific CD8^+^ T cells accumulate in the brain predominantly after sporozoite infection. The experimental setup used was identical to the one described for the cross-presentation assay. Lymphocytes derived from brains and spleens of infected and naïve mice were used to perform an IFN-γ ELISpot assay. Brain-infiltrating lymphocytes and splenocytes from infected mice were cultured together with antigen-presenting cells (APCs) (naïve splenocytes) that were previously pulsed with a pool of *Pb*maLS_05 peptides or Pb1 peptide of GAP50. APCs pulsed without peptides were cultured together with lymphocytes, as control. Statistical significance was determined by One-way ANOVA with Bonferonni’s correction for the spleens and Kruskal–Wallis with Dunn’s multiple comparison for the brains (***p < 0.0001, **p < 0.001, ns; not significant). The results shown with median are pooled from two independent experiments (*n* = 10–12 mice per group; *n* = 5 mice for KO-iRBC). **(B)** Experimental setup to test *in vivo* cytotoxicity of *Pb*maLS_05-specific CD8^+^ T cells. 5 *Pb*ANKA iRBC-infected and 4 *Pb*ANKA sporozoite-infected mice were injected with *Pb*maLS_05 peptides; 2 iRBC-infected and 3 sporozoite-infected mice were injected with the Pb1 (GAP50) peptide and 5 mice were used as naïve controls. **(C)** *Pb*maLS_05-specific CD8^+^ T cells are responsible for the development of ECM in sporozoite-infected mice. Groups of mice were infected according to the described schedule **(B)** and treated with antimalarial drugs (CQ + ART) prior to injections with peptides. One day after the peptide injection, all mice were challenged with two injections of folic acid, 1 h apart. The bar graph represents percentage survival in each group, after 90 min. The data shown are representative of a single experiment.

In spite of a statistical difference in the levels of cross-presentation of Pb1 by the brain endothelium after sporozoite infection, it was very unlikely that this factor alone fully accounted for the complete absence of ECM in KO-infected mice, especially given the overlap in data points with WT-infected mice. Moreover, there was a 10-fold difference in the level of Pb1 cross-presentation between KO-sporozoite and KO-iRBC-infected mice (Figure 8). Since the development of ECM is in part an adaptive immune response, it is plausible that the antigens capable of eliciting an immune response might differ between iRBC and sporozoite infections. During sporozoite infection, the immune system is first exposed to sporozoite and liver stage antigens and early priming of T cells recognizing these antigens could bias the subsequent response during blood-stage infection. To determine if the differences between KO-sporozoite and KO-iRBC infections were caused due to changes in the immune-dominance of antigens, we considered if *Pb*maLS_05 could itself be immunogenic. To investigate whether CD8^+^ T cells recognizing *Pb*maLS_05 were primed during an infection, we predicted CD8^+^ T cell epitopes for *Pb*maLS_05 and tested synthesized peptides in a cultured ELISpot assay. As determined from the spot counts representing IFN-γ responses, *Pb*maLS_05 was predominantly recognized by brain-infiltrating lymphocytes of WT-infected mice after sporozoite injection (Figure 9A) and to a lesser extent after iRBC infection (Figure 9A). Furthermore, the absence of a *Pb*maLS_05-specific response in the brains of KO sporozoite-infected mice confirmed the deletion of *Pb*maLS_05, thus providing an additional negative control. We detected a minor response to *Pb*maLS_05 in the spleens of both sporozoite and iRBC-infected mice (Figure 9A), despite a prominent response to the Pb1 epitope of GAP50 (Figure S7 in Supplementary Material), indicating that the spleen might not be the primary site for priming of *Pb*maLS_05-specific CD8^+^ T cells.

To test if these *Pb*maLS_05-specific cells contributed to the development of ECM after both sporozoite and iRBC infections, we used a similar experimental setup as previously described (45). To investigate if *Pb*maLS_05-specific CD8^+^ T cells were capable of inducing rupture of the blood–brain barrier *in vivo* (Figure 9B), we infected groups of mice with WT sporozoites or iRBCs. On day 5 p.i. for the iRBC-infected group and day 7 p.i. for the sporozoite-infected group, we treated mice twice with chloroquine (CQ) and artesunate (art), to clear peripheral parasitemia and reduce any circulating parasite antigen that could be cross-presented on the MHC I complex of brain endothelial cells. After the drug treatment, we artificially loaded MHC I complexes including those in the brain microvasculature with a pool of *Pb*maLS_05 peptides through repeated intravenous injections of soluble peptide. Following the last peptide injection, all mice were challenged with folic acid, a neurotoxin, to determine if the blood–brain barrier had been compromised or not. To evaluate the efficiency of the procedure, we included an additional group of sporozoite and iRBC-infected mice where we injected an equal amount of the Pb1 peptide, instead of *Pb*maLS_05.

Both sporozoite and iRBC-infected mice injected with the Pb1 peptide convulsed and died after the folic acid injection, thus confirming previous reports that proposed an immunodominant role for Pb1 in ECM [Figure 9C (45)]. Interestingly, only sporozoite-infected mice treated with *Pb*maLS_05 peptides succumbed to cerebral symptoms after the folic acid injection, in contrast to the iRBC-infected group where none of the mice developed ECM symptoms. These data, therefore, implied that *Pb*maLS_05-specific CD8^+^ T cells contributed to permeabilization of the blood–brain barrier only after sporozoite infection (Figure 9C).

In summary, these data support a dual role for *Pb*maLS_05 in the development of ECM, specific to sporozoite and iRBC-induced infections, and implicate a direct role for pre-erythrocytic stages in the development of cerebral symptoms.

## Discussion

In this study, we investigated a well-conserved *Plasmodium* gene, *Pb*maLS_05 that is targeted to the apicoplast of intra-erythrocytic and late intra-hepatic parasites. Deletion of the endogenous *Pb*maLS_05 had little impact on life cycle progression of the parasite but affected the development of ECM in mice. While the abrogation of ECM was consistent after both sporozoite and iRBC infections, parasite sequestration and the host immune response differed significantly between the two modes of infection. Moreover, *Pb*maLS_05-specific CD8^+^ T cells were found to contribute to the development of ECM after sporozoite but not after iRBC infection. Our results, therefore, implicate a role for pre-erythrocytic antigens like *Pb*maLS_05 in the modulation of host immune responses that contribute to the development of ECM.

The apicoplast of *Plasmodium* parasites encodes different anabolic products that are essential for parasite survival and propagation through the life cycle (79). Despite the localization in the apicoplast, *PbmaLS_05* (−) parasites were capable of completing the life-cycle, thus implying a redundant or non-essential role for *Pb*maLS_05 in functions of the apicoplast. Based on the timing of expression of *Pb*maLS_05 in the apicoplast of both late liver- and blood-stage schizonts, we hypothesized that *Pb*maLS_05 might play a role in apicoplast segregation. Previous studies have shown that a co-ordinated division of the apicoplast from mid to late stages of nuclear division was required to ensure that every daughter cell inherits one apicoplast (70, 80). In fact, disruption of apicoplast segregation by transfection with an ACP-GFP-mRON1 plasmid in *Toxoplasma gondii* parasites was shown to result in a “delayed death” phenotype as seen with antibiotics that affect the apicoplast such as azithromycin (58). It was observed that parasites devoid of an apicoplast survived for a short-term, while those containing a single large apicoplast had reduced growth rates in comparison to un-transfected controls (81). It is, therefore, conceivable that a defect in apicoplast segregation in *PbmaLS_05* (−) parasites was responsible for the modest reduction in growth rates in the blood of infected mice. However, neither apicoplast branching nor division was abnormal in *PbmaLS_05* (−) parasites, thus excluding our hypothesis. Interestingly, the reduction in *PbmaLS_05* (−) parasites in the blood were not supported by increased parasite sequestration in the spleen (data not shown), suggesting that defective sequestration might not account for the reduction in circulating parasitemia in KO-infected mice. To determine if the growth defect of *PbmaLS_05* (−) parasites was due to reduced multiplication rates, we analyzed the parasite growth kinetics in KO-iRBC-infected mice, through a mathematical model. Interestingly, the model suggested that deletion of *PbmaLS_05* had a modest effect on the ability of parasites to develop within reticulocytes during the initial stages of infection (82). Therefore, it is plausible that a minor population of KO parasites that fail to develop within reticulocytes are cleared by the spleen and elicit an immune response that protects against ECM. However, an analysis of how parasite reticulocyte preference affects the immunopathogenesis of ECM is outstanding.

The spleen is instrumental in both clearance of dead or damaged parasites from circulation and regulation of parasitemia in the blood (15). Phagocytosis of iRBCs or free merozoites by splenic DCs begins soon after infection and primes the T cell response that results in ECM. The presentation of parasite antigens such as GAP50 (Pb1 epitope, SQLLNAKYL) by splenic CD11c^high^ Clec9A^+^ DCs primes both CD4^+^ and CD8^+^ T cells which then migrate to the brain and other organs *via* chemotaxis (76). Within the brain, cytotoxic CD8^+^ T cells recognize epitopes such as Pb1 that are cross-presented by the endothelium and secrete IFN-γ, granzyme B, and perforin, which contribute to inflammation and blood–brain barrier disruption (44, 46, 83). Previous experiments have shown that treatment of mice with chloroquine or artesunate reduces the parasite load in peripheral tissues and thus prevents cross-presentation and ensuing ECM symptoms (35, 45). The absence of ECM following an infection with *PbmaLS_05* (−) iRBCs could, therefore, be attributed to a reduction in peripheral parasitemia and reduced parasite sequestration in the brains of infected mice. Furthermore, the reduced sequestration of activated CD8^+^ T cells in the brains of mice following an infection with *PbmaLS_05* (−) iRBCs is also consistent with studies showing a requirement of activated CD8^+^ T cells for ECM to occur (75). In contrast, we observed no difference in parasite load in the brains of *PbmaLS_05* (−) sporozoite-infected mice, in spite of lower numbers of parasites in circulation. Moreover, leukocyte infiltration within the brains of *PbmaLS_05* (−) sporozoite-infected mice was also unaffected when compared WT sporozoite-infected mice, despite the difference in ECM outcome.

These dissimilarities in the host immune response between iRBC and sporozoite infections can either be attributed to modulation of T cell responses by pre-erythrocytic stages, or modifications in the parasite inoculum following sporozoite or blood-stage infection. As previously shown by Spence et al., differences in gene regulation of variant surface antigens exist between serially passaged and mosquito transmitted *P. chabaudi chabaudi* parasites. In fact, sporozoite inoculation was found to attenuate parasite growth and virulence subsequently modifying the host immune response (48, 84). Therefore, it is likely that vector transmission alters gene expression in a way that broadens the antigenic repertoire of sporozoite-induced blood-stage parasites in contrast to blood-passaged infections. In other studies, sporozoite-infected mice were shown to develop lower parasitemia with reduced frequencies and activation levels of CD8^+^ T cells in the brain, compared to those infected with iRBC (85, 86). The interesting aspect, however, is that despite the existing differences in immune responses, both sporozoites and intra-erythrocytic parasite stages are capable of inducing ECM in susceptible mice. Moreover, sporozoite-infected mice develop ECM in spite of the lower frequencies of CD8^+^ T cells in brains suggesting that T cell priming in the spleen is more efficient in comparison to blood-stage-induced infection (78). It is, therefore, possible that the mechanism of protection from ECM after an infection with *PbmaLS_05* (−) sporozoites originates from a modified immune response against virulent antigens or altered priming of T cells in response to different antigens (87, 88).

Interestingly, we found equal numbers of Pb1-specific CD8^+^ IFN-γ^+^ T cells in the brains of WT and *PbmaLS_05* (−) infected mice after sporozoite infection, suggesting no difference in priming of antigen-specific CD8^+^ T cells in the spleen. In fact, a study by Howland et al. identified equal numbers of infiltrated Pb1-specific CD8^+^ T cells in the brains of mice infected with ECM and non-ECM causing parasite strains (45). A study by Shaw et al. also corroborated observations made by Howland et al., thus confirming that the presence of CD8^+^ T cells in the brain alone is insufficient to cause ECM (78). Both studies demonstrated the need for cross-presentation of parasite-specific antigens by the activated endothelium and engagement of antigen-specific T cells with their corresponding APC in the brain microvasculature. The Pb1 peptide sequence of GAP50 was one dominant epitope identified as being cross-presented by endothelial cells (45, 78). Since *PbmaLS_05* (−) sporozoite-infected mice did not develop ECM, despite the presence of Pb1-specific IFN-γ^+^ CD8^+^ T cells in the brain microvasculature, we reasoned that perhaps the absence of ECM could be due to a lack of cross-presentation of antigens by the brain endothelium. Indeed, the cross-presentation of Pb1 after sporozoite infection, by brain microvessels isolated from *PbmaLS_05* (−)-infected mice was lower than that of WT-infected mice, though not as significant as what we observed after an iRBC infection. The source of cross-presented Pb1 was previously identified as merozoites that were phagocytosed by brain endothelial cells (47). Therefore, it is plausible that impairment in phagocytosis of merozoites was responsible for the reduced cross-presentation of Pb1 in KO-sporozoite-infected mice, particularly since parasite sequestration in the brain was similar to that of WT-sporozoite-infected mice.

Interestingly, we noticed a 10-fold difference in the level of cross-presentation of Pb1 between KO-sporozoite and KO-iRBC-infected mice, despite comparable parasite sequestration in the brain. Assuming that deletion of *PbmaLS_05* affects cross-presentation of parasite antigens in the same manner after both sporozoite and iRBC infection, it is improbable that this disparity in Pb1 cross-presentation was caused due to variations in the process of phagocytosis and antigen presentation between both infection groups. Interestingly, a recent study characterizing CD4^+^ T cell epitopes in *P. berghei* reported a change in the immunodominance of antigens depending on whether infection was initiated by sporozoites or iRBCs (89). It is, therefore, likely that the hierarchy of cross-presented antigens contributing to ECM development also differs between sporozoite and iRBC infections. While Pb1 was identified as a immunodominant epitope, another study by Poh et al. identified additional CD8^+^ T cells epitopes that influenced ECM outcome. The authors moreover contended that damage to the blood–brain barrier results from a synergistic effect of T cells with differing antigenic specificities (88). It is interesting to note that Pb1 and other CD8^+^ T cell epitopes influencing the outcome of ECM were identified post-infection with iRBCs (45, 87, 88). Therefore, it is possible that their influence might be dampened by the availability of a broader spectrum of antigens after sporozoite infection.

Since *Pb*maLS_05 was expressed during both sporozoite and iRBC infections, we hypothesized that perhaps *Pb*maLS_05 in addition to Pb1 is required for ECM development after sporozoite infection. Indeed brain-infiltrating lymphocytes from the majority of WT-sporozoite-infected mice responded to the stimulation with *Pb*maLS_05 peptides in significant numbers. Brain-infiltrating lymphocytes from mice infected with WT iRBCs also produced IFN-γ in response to *Pb*maLS_05, although to a lesser extent than after sporozoite infection. Despite no significant difference in the numbers of brain *Pb*maLS_05-specifc CD8^+^ T cells, only sporozoite- and not iRBC-infected mice displayed signs of blood–brain barrier disruption, after *Pb*maLS_05 peptides were injected to load the endothelium, thus concluding that only sporozoite-induced *Pb*maLS_05-specific CD8^+^ T cells contributed to the development of ECM *in vivo*.

Surprisingly, we did not observe a significant IFN-γ response to *Pb*maLS_05 in the spleens of WT-sporozoite and iRBC-infected mice, in spite of a prominent response to Pb1, thus arguing against the priming of *Pb*maLS_05-specific CD8^+^ T cells within the spleen. Given that *Pb*maLS_05 is expressed in both late pre-erythrocytic and intra-erythrocytic stages, it is plausible that *Pb*maLS_05-specific CD8^+^ T cells are first primed during the pre-erythrocytic stage and then undergo expansion upon blood-stage infection. Nevertheless, CD8^+^ T cells specific to *Pb*maLS_05 disrupted the blood–brain barrier only after sporozoite infection, thus implying that the antigen responsible for CD8^+^ T cell cytotoxicity is probably presented during the sporozoite and not iRBC stage of infection. Although the data implies that *Pb*maLS_05 might be cross-presented by the brain endothelium after sporozoite infection, it warrants further investigation and identification of the correct epitope that is cross-presented by brain endothelial cells during an infection.

On the basis of these data, we can propose that *Pb*maLS_05 contributes to the development of ECM in more than one way. Given the observed blood-stage growth defect after sporozoite and iRBC infection, it is possible that *Pb*maLS_05 plays a role in parasite viability, possibly by contributing to a function of the apicoplast that renders parasites capable of developing within reticulocytes. In addition, it is also possible that *Pb*maLS_05 functions as an antigen that directly influences the host immune response after sporozoite infection, presumably by first being presented during the pre-erythrocytic stage of development and then by microvessels in the brain.

In conclusion, we have identified a cross-stage antigen, *Pb*maLS_05, that contributes to the development of ECM after both sporozoite and iRBC infections. Since the importance of cross-stage immunity is gaining relevance in malaria vaccine development, immune mechanisms eliciting responses against shared antigenic targets, particularly those shared between late liver and blood-stages, are currently being considered (90, 91). In the process of determining the function of one such antigen, *Pb*maLS_05, we uncovered a role for pre-erythrocytic parasite development in the development of cerebral symptoms. Our findings, therefore, have implications for therapeutic strategies to combat cerebral malaria, especially those focusing exclusively on either intra-erythrocytic or pre-erythrocytic stages of the parasite life cycle.

## Ethics Statement

All experimental animal procedures were performed in accordance with standard guidelines as set by regulations concerning FELASA category B and GV-SOLAS. Animal experiments were approved by the German authorities (Regierungspräsidium Karlsruhe, Germany), 1 8 Abs. 1 Tierschutzgesetz (TierSchG) under the license G-258/12. 6- to 8-week-old female C57BL/6J and NMRI mice were purchased from Janvier, France and kept under specific pathogen-free (SPF) conditions at the animal facility (IBF) of the University of Heidelberg.

## Author Contributions

PF and AKM proposed the scientific hypothesis and organized the study. PF performed all the experiments unless specified otherwise. SH performed the iRBC cross-presentation assay, KH and RF performed the IVIS bioluminescence imaging. KO performed the ACP-GFP co-staining. AH and MB assisted with MRI measurements. AH analyzed histological sections. PF, KH, and MC performed all the immunological experiments. PF, SH, AH, KH, and LR analyzed and interpreted the data. PF and AKM wrote the paper. PF, KH, SH, LR, and AKM edited and finalized the manuscript. All authors commented on the paper.

## Conflict of Interest Statement

The authors declare that the research was conducted in the absence of any commercial or financial relationships that could be construed as a potential conflict of interest.

## Footnotes

^1^www.syfpeithi.de (Accessed: April, 2016).

^2^http://www-bimas.cit.nih.gov/ (Accessed: April, 2016).
