# Supplementary material for: A Plasmodium Cross-Stage Antigen Contributes to the Development of Experimental Cerebral Malaria
Source: Front Immunol. 2018 Aug 14;9:1875. doi: 10.3389/fimmu.2018.01875 (PMC6102508; doi:10.3389/fimmu.2018.01875)
Supplement: Supplementary file 1 [file Presentation_1.PDF]

Supplementary Material

A *Plasmodium* cross-stage antigen contributes to the development of Experimental Cerebral Malaria

Priyanka Fernandes[1#], Shanshan W. Howland[2], Kirsten Heiss[1,4], Angelika Hoffmann[3], Maria A. Hernández-Castañeda[1], Klára Obrová[1], Roland Frank[1], Philipp Wiedemann[5], Martin Bendzus[2], Laurent Renia[3] and Ann-Kristin Mueller[1,4*]

[1]Centre for Infectious Diseases, Parasitology Unit, University Hospital Heidelberg, D-69120 Heidelberg, Germany.

[2]Singapore Immunology Network, Agency for Science, Technology and Research (A*STAR), Singapore, Singapore

[3]Department of Neuroradiology, Heidelberg University Hospital, Germany and Division of Experimental Radiology, Department of Neuroradiology, Heidelberg University Hospital, Germany

[4]German Centre for Infection Research (DZIF), Heidelberg, Germany.

[5]Department of Biotechnology, Mannheim University of Applied Sciences, D 68163 Mannheim, Germany

* **Correspondence:** Corresponding Author: ann-kristin.mueller@uni-heidelberg.de

Table S1: List of primers

*PbmaLS*_05 (-) vector primers

*Pb*AgA_5'UTR_ forward	5'-ATC CGC GGG GCA TTA TTA GAT GTC ATA GGA GCG-3'
*Pb*AgA_5'UTR_ reverse	5'-ATA CAT ATG GGA TTA AAT ATA CAC ACG CAC AAC G-3'
*Pb*AgA_3'UTR_ forward	5'- ATT AAG CTT CGA GTA TTG CTT ACG TTT AAA TTG ATA GAG-3'
*Pb*AgA_3'UTR_ reverse	5'- ATC TCG AGG CCC TAA ATA GGA ATA ATA ATG CAA AAT GC-3'
*Pb*AgA_Seq_5'UTR_ forward	5'-CGG AAA GCA GCA ATA ACA CTA CTA C-3'
5'int_3'_rev	5'-GAT CCT TAC TTG TAC AGC-3'
*Pb*AgA_Seq_3' UTR	5'- CGG CGA AAT TAT ATT GCT ACC GT-3'
*Tg*DHFR/TS_reverse	5'-GCA GTT GAT TTG TTT GAA AGA ATG TC-3'
WT_forward	5'-GCA AAG GCG GAG AAA TAC C-3'
WT_reverse	5'-CAC CCG TAG TAG CAT CTT CC-3'

*Pb*maLS_05 CT EGFP tagging vector

*Pb*AgA_3'UTR_ forward	5'- ATT AAG CTT CGA GTA TTG CTT ACG TTT AAA TTG ATA GAG-3'
*Pb*AgA_3'UTR_ reverse	5'- ATC TCG AGG CCC TAA ATA GGA ATA ATA ATG CAA AAT GC-3'
*Pb*AgA_3'end_ forward	5'-ATC CGC GGC GCA ATT GCA AGA AAT TGC TAT GT-3'
*Pb*AgA_3'end_ reverse	5'-ATA TCT AGA ATA GTG TTT CGT TTT TTT TAA AAT CAT ATT GGC C-3'

EGFP_forward	5’- GCA CTA GTG CCG CCG CCG TGA GCA AGG GCG AGG AGC TG-3’
EGFP_reverse	5’- GCG GAT CCT TAC TTG TAC AGC TCG TCC ATG CCG AG-3’
5’ tag_sequencing _forward	5’-GCA AAA GAT TCT TTA TGG ATA ATA GGG G-3’
b3D+ reverse	5’-CCT TGC TCA TTT ACC TGC TAA TAC GAT TGC-3’
*Tg*DHFR/TS_reverse	5’- CGG CGA AAT TAT ATT GCT ACC GT-3’
3’ sequencing_reverse	5’- GCA GTT GAT TTG TTT GAA AGA ATG TC-3’

Transcriptional analysis

PP1_forward	5’-GCG GTG GAA GAC GGA ATC AAG AAG G-3’
PP1_reverse	5’-GAG GGG AAA GGG AAT ATA TAG C-3’
PP2_forward	5’-GCT ATA TAT TCC CTT TCC CCT CTT ATT TAT AGC-3’
PP2_reverse	5’-CAA TTG GTA ATA CTT GTT CAA CTC-3’
PP3_forward	5’-GCA ACT TCC ACA ATG TGC TCA TG-3’
PP3_reverse	5’-CGT AAA AGT CCC ATT CTA GAA ACT CCT GC-3’
PP4_forward	5’-CAA CAC GCT TTA GAA ATG AGG ACG-3’
PP4_reverse	5’-CGT AAA CTA TCA CTA CCA CCT TC-3’
PP5_forward	5’-CTG GTA GTG CAT CGC CAA TTT TAG-3’
PP5_reverse	5’-GCA CTT GAG ATT GGT ATG GGC AAA TAA TAC C-3’

Quantitative Reverse Transcriptase Real Time analysis (qRRT-PCR)

Mouse GAPDH_Forward	5’-TTG ATG GCA ACA ATC TCC AC-3’
Mouse GAPDH_reverse	5’-CGT CCC GTA GAC AAA ATG GT-3’
Pb 18S rRNA_forward	5’-AAG CAT TAA ATA AAG CGA ATA CAT CCT TAC-3’
Pb 18S rRNA_reverse	5’-GGA GAT TGG TTT TGA CGT TTA TGT G-3’

Table S2: List of *Pb*maLS_05 specific CD8 T cell epitope peptides

Name	Sequence
*Pb*maLS_05_K8L	KLDYYEKL
*Pb*maLS_05_I8I	ILYFYNKI
*Pb*maLS_05_E8L	ENIEFEYL
maLSA_1568	TSLENLKPM
maLSA_395	SIFLYWIKL
maLSA_64	VVYFFYTNV
maLS05_827	LTYVFNTI
maLS05_147	SVIKNDENL
maLS05_1562	ASNENKTSL

Figure S1:

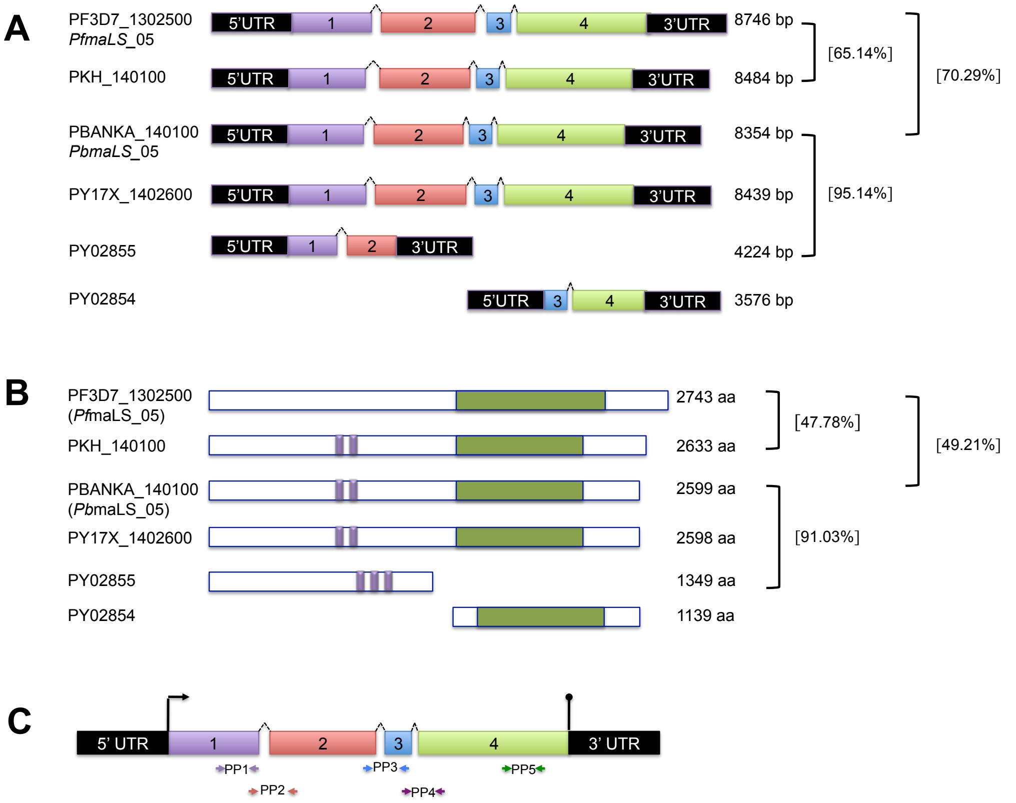

Developmental stage	PP1	PP2	PP3	PP4	PP5
Liver stages (24 h.p.i)	+	+	-	-	-
Liver stages (48 h.p.i)	+	+	+	+	+
Liver stages (63 h.p.i)	+	+	+	+	+
Mixed Blood stages	+	+	+	+	+
Schizonts (Blood)	+	+	+	+	+
Gametocytes	+	+	+	+	+
Mid-gut sporozoites	+	+	-	+	+
Salivary gland sporozoites	+	+	+	+	+

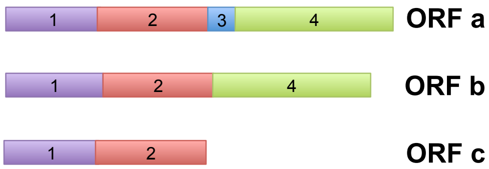

Supplementary Figure 1. *maLS_05* is conserved in *Plasmodium* species both at the genomic and proteomic level and transcribed throughout the parasite life cycle

(A) Alignments of gene sequences of *P. falciparum* (PF3D7_1302500), *P. knowlesi* (PKH_140100), *P. berghei* (PBANKA_140100), *P. yoelii yoelii 17X* (PY17X_1402600), *P. yoelii yoelii 17XNL*

(PY02854 and PY02855) was done using the ClustalW tool from EMBL-EBI. **(B)** Alignments of protein sequences of PF3D7_1302500, PKH_140100, PBANKA_140100, PY17X_1402600, PY02855 and PY02854 are displayed along with % sequence similarity in brackets. The *PfmaLS*_05 orthologue in *P. yoelii yoelii 17XNL* is split into two open reading frames (ORF) of which the function of PY02854 is annotated as a SEN-1 related protein (PlasmoDB), while the function of PY02855 is unknown. All orthologues of *Pf*maLS_05 contain conserved predicted domains (transmembrane domains are shown in purple and a P loop containing nucleoside triphosphate hydrolase shown in green). **(C)** The *PbmaLS*_05 gene consists of four exons and has a predicted mRNA size of 7800 bp. RT-PCR analysis using primers depicted in the schematic, revealed the presence of distinct alternatively spliced variants of *Pb*maLS_05 in mid-gut sporozoites and early liver stages (24 h.p.i.). The plus and minus signs indicate presence and absence of transcript respectively. For convenience, the full-length isoform was termed 'ORF a' and the suggested spliced isoforms in mid-gut sporozoites and liver stages 24 h.p.i 'ORF b' and 'ORF c' respectively.

Figure S2:

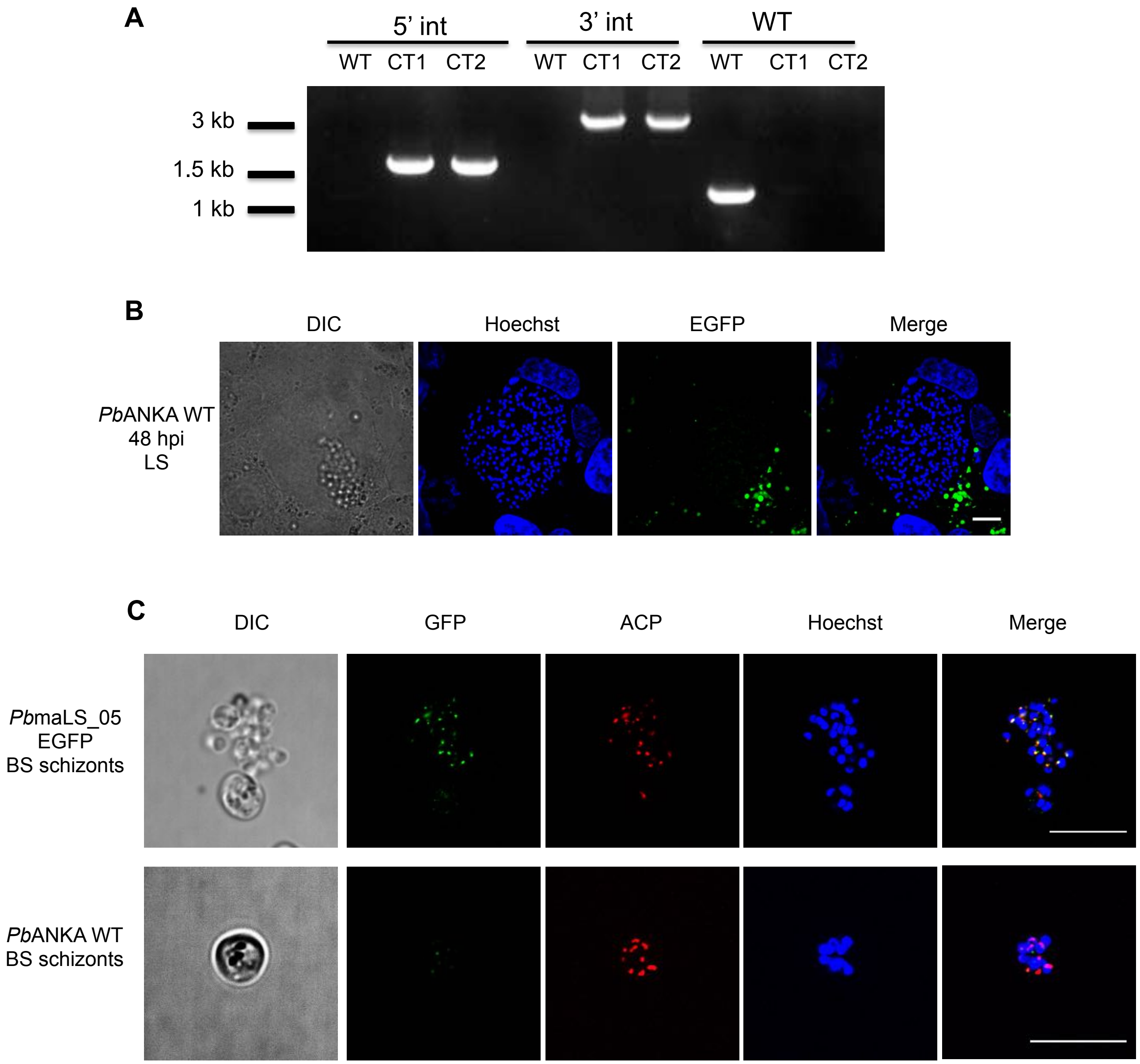

Supplementary Figure 2. Genotyping of clonal *Pb*maLS_05 CT EGFP parasites and localisation of *Pb*maLS_05 in the parasite.

(A) *Pb*maLS_05 CT EGFP transfected parasites were cloned out using limiting dilution. Stable integration of the tagging construct was confirmed by live microscopy and PCR using primers flanking the integration sites. Clonal populations of *Pb*maLS_05 EGFP parasites were passaged through the life cycle and analysed for expression. **(B)** No expression above background in *Pb*ANKA WT liver stages, confirm the expression and localisation of *Pb*maLS_05 as seen in *Pb*maLS_05 EGFP parasites. *In vitro*

liver stages of *Pb*ANKA WT parasites were imaged live using a spinning disc confocal microscope. The nuclei were stained with Hoechst briefly before imaging. Scale bar =10 µm. **(C)** *Pb*maLS_05 localises to the apicoplast of blood-stage schizonts. *Pb*maLS_05 EGFP blood-stage schizonts co-stained with GFP and ACP antibodies *Pb*maLS_05 EGFP showed co-localisation of both antibodies, thus confirming that *Pb*maLS_05 was targeted to the apicoplast of blood-stage schizonts. No signal above background in the GFP channel was visible for *Pb*ANKA WT blood-stage schizonts. Scale bar = 10 µm.

Figure S3:

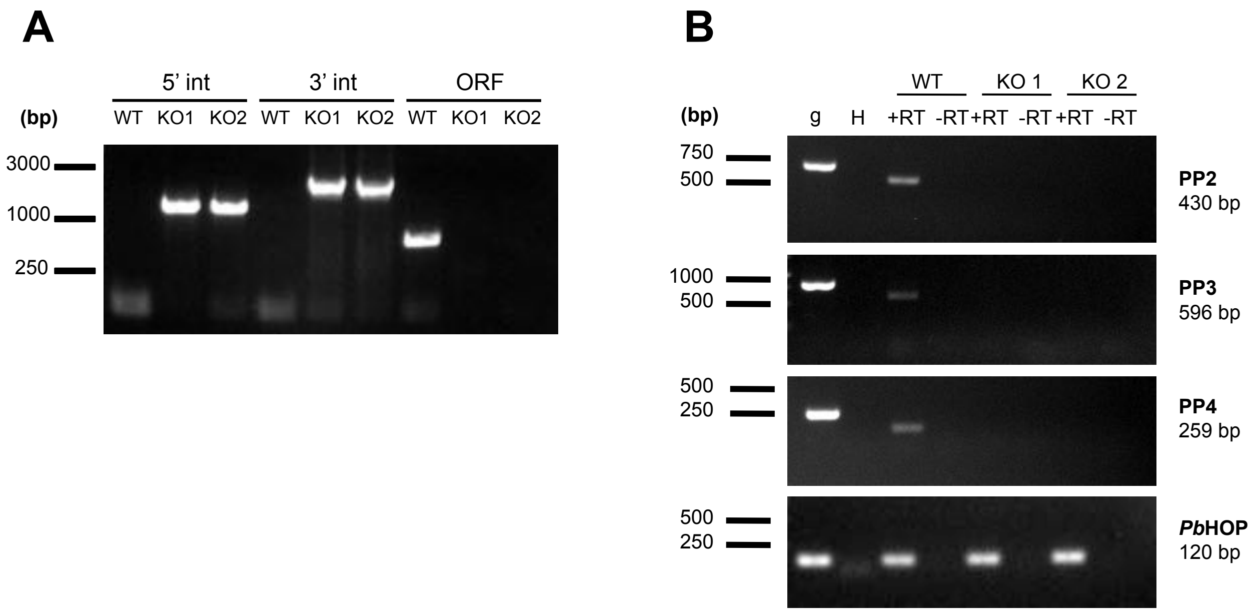

Supplementary Figure 3. Genotyping PCR for *PbmaLS_05* (-) parasites and verification of clonal populations.

(A) Stable integration of the *PbmaLS_05* (-) construct was verified by PCR primers flanking the integration sites for both the 5' and 3' ends. Absence of a WT signal in the PCR amplifying a 500 bp fragment of the WT genomic locus verified the purity of clonal populations of *PbmaLS_05* (-) parasites. **(B)** Absence of any residual WT transcript was verified by exon specific RT-PCR on blood stage schizont cDNA.

Figure S4:

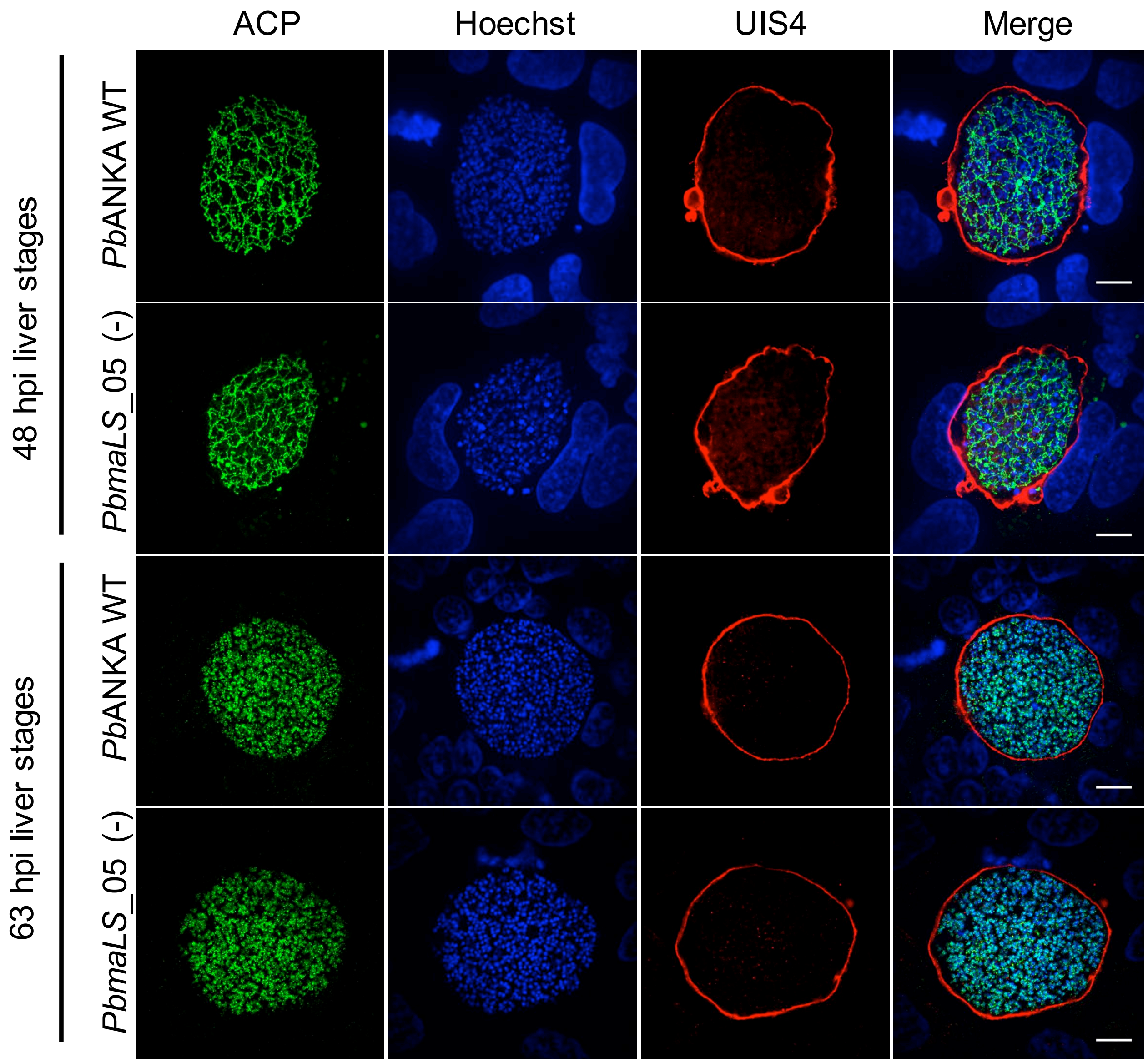

Supplementary Figure 4. Apicoplast branching and inheritance is indistinguishable between WT and KO parasites.

HuH7 cells were infected with *Pb*ANKA WT or *PbmaLS_05* (-) sporozoites and fixed at 48 h.p.i. or 63 h.p.i. Fixed cells were incubated with antibodies against the apicoplast and parasitophorous vacuole membrane (PVM; UIS4) while the nuclei were stained with Hoechst. Images of infected hepatocytes were acquired using a spinning disc confocal microscope and processed using ImageJ. Scale bar = 10 μm.

Figure S5:

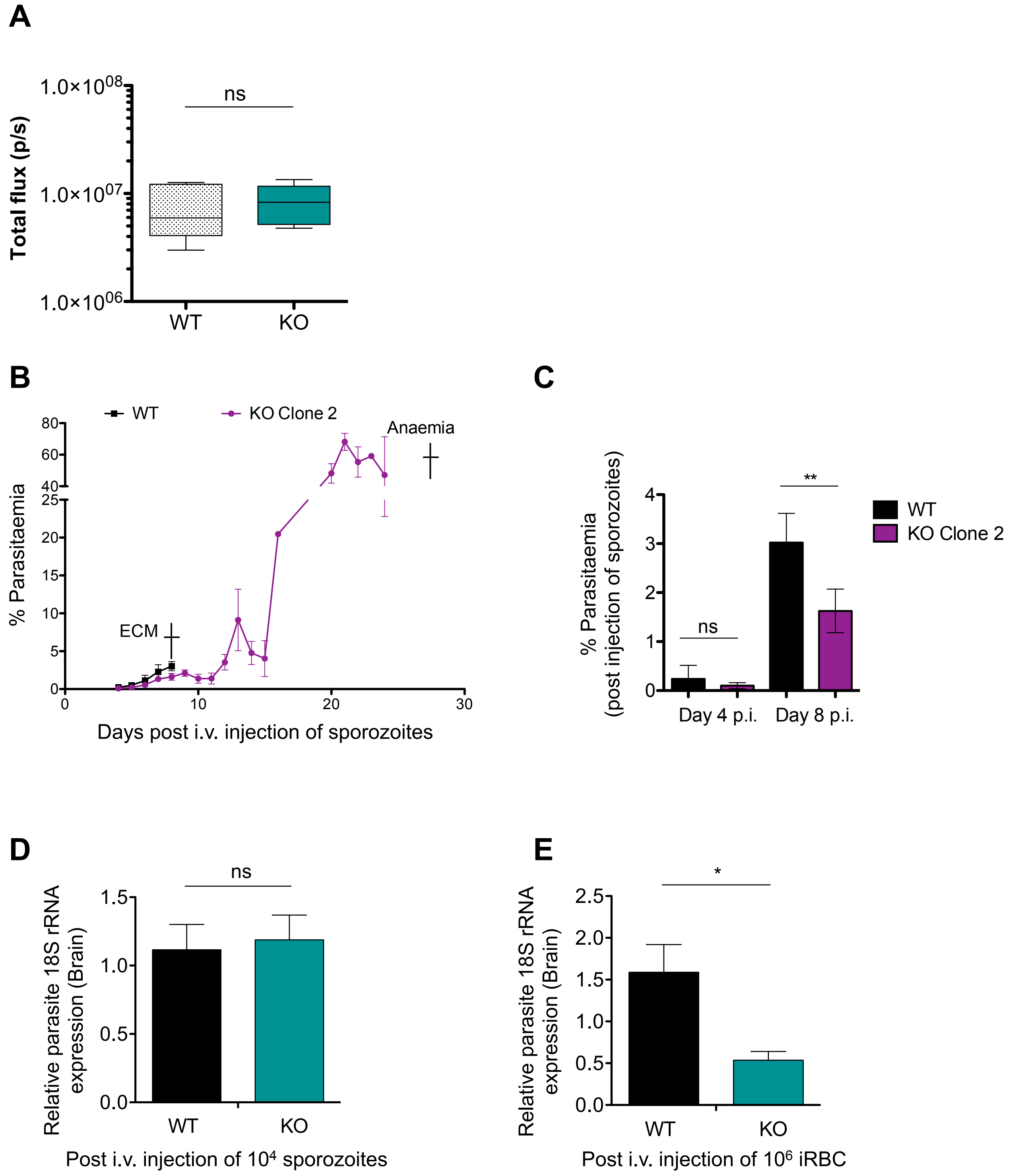

Supplementary Figure 5. The non-ECM phenotype in KO sporozoite-infected mice was also observed for an independent clone.

(A) Groups of 5 C57BL/6J mice were intravenously injected with 10^4 *Pb*GFP Luc_{con} WT or *PbmaLS_05* (-) GFP Luc_{con} sporozoites and then imaged at 72 hours post infection, using the IVIS imaging system. Prior to imaging, each mouse was injected intraperitoneally with luciferin and the bioluminescence recorded as total flux (photons/second). The Mann-Whitney *U* test was used to determine statistical significance. (ns, not significant). **(B)** In contrast to WT-infected mice, all KO sporozoite-infected mice did not develop ECM and were eventually sacrificed when mice were anaemic due to hyperparasitaemia. **(C)** The parasitaemia on day 8 p.i. was significantly lower in KO-infected mice, despite no difference to WT sporozoite-infected mice at day 4 p.i. Values represent Mean ± SD. The Mann-Whitney *U* test was used to determine statistical significance. (**p<0.001; ns, not significant). **(D)** Quantification of parasite load in brains of WT and KO infected mice injected with sporozoites, by qRT-PCR analysis. Groups of 5 C57BL/6J mice were intravenously injected with 10^4 sporozoites of WT or KO and sacrificed when WT-infected mice showed signs of ECM. Brains were harvested after perfusion and parasite load in the brain quantified by qRT-PCR for parasite 18SrRNA transcripts. Analysis of the brains of WT and KO sporozoite-infected mice showed comparable numbers of parasite 18S rRNA transcripts in the brain, despite the difference in disease outcome. **(E)** Quantification of parasite load in brains of WT and KO infected mice injected with sporozoites, by qRT-PCR analysis. Groups of 5 C57BL/6J mice were intravenously injected with 10^6 iRBCs of WT or KO and sacrificed when WT-infected mice showed signs of ECM. Relative quantification of parasite 18S rRNA transcripts after iRBC infection, suggested lower parasite numbers in the brains of KO-infected mice. Relative expression of *Pb*18S rRNA quantified by qRT-PCR was normalised to mouse GAPDH (Statistical significance was determined by the Student's *t* test, **p<0.001; *p<0.01; ns, not significant).

Figure S6:

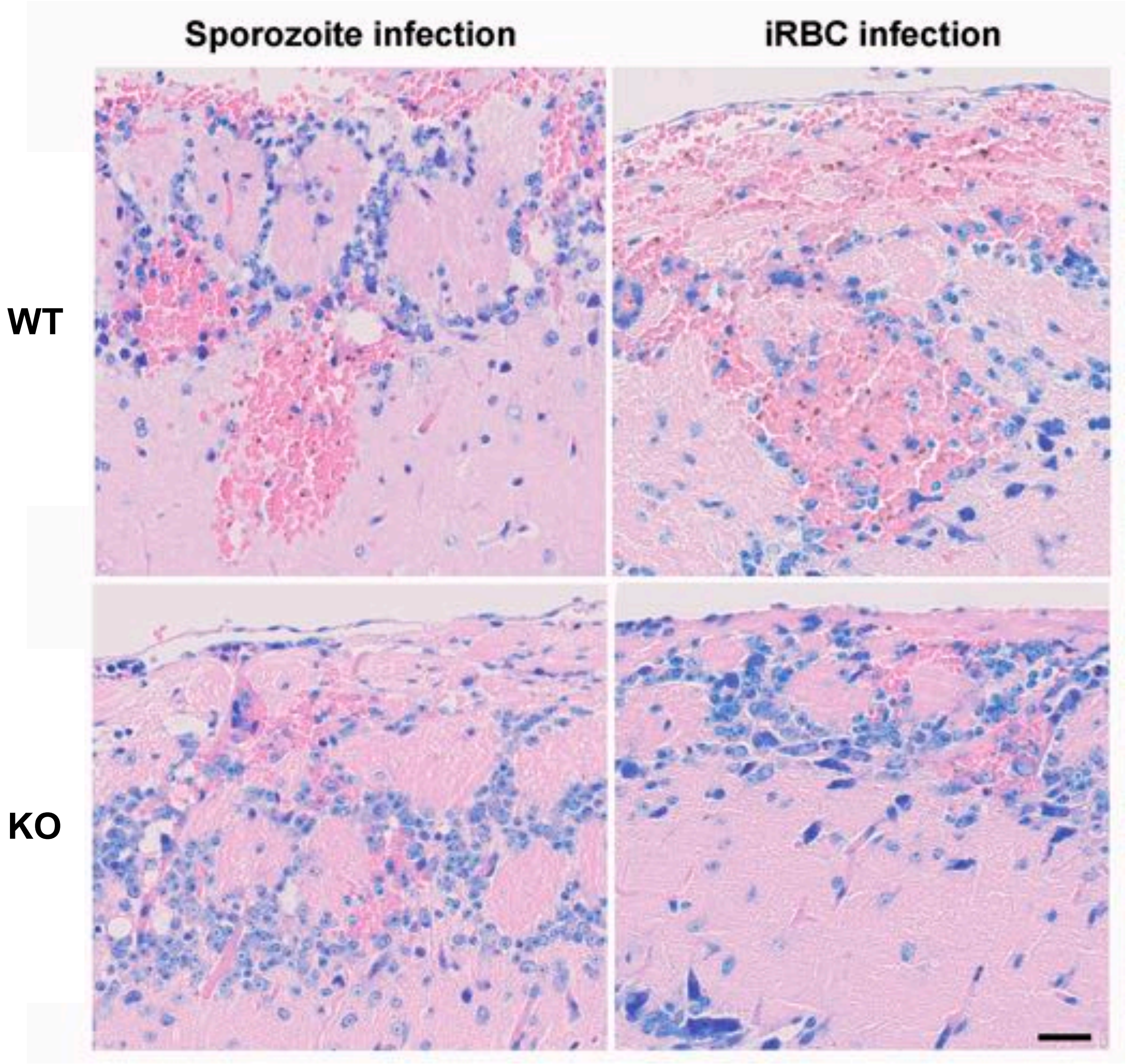

Supplementary Figure 6. Microhemorrhages in the olfactory bulb on histological sections. Microhemorrhages were confirmed from histological sections. Exemplary images of Giemsa stained olfactory bulbs are shown. In *Pb*ANKA WT-infected mice severe microhemorraghing was evident (first row), while only few microhemorrhages were seen in the KO (second row). Scale bar =50µm.

Figure S7:

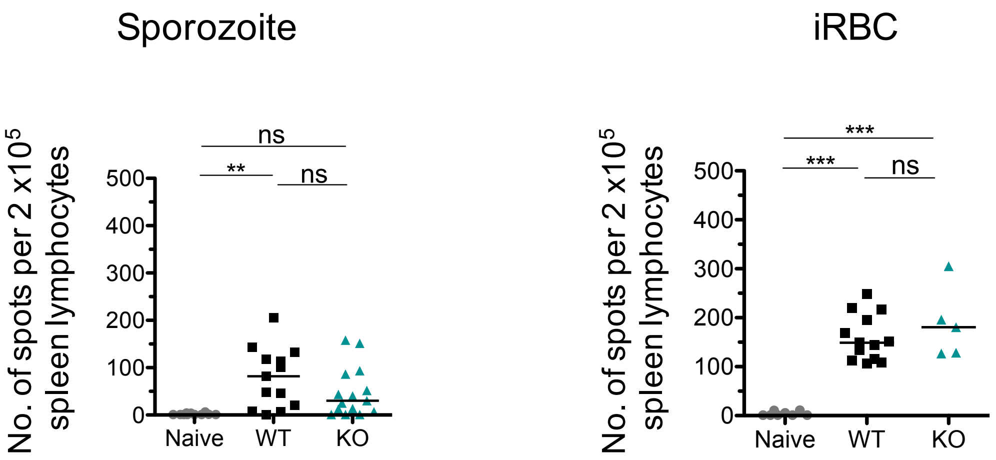

Supplementary Figure 7. IFN-γ ELISpot results for Pb1 (GAP50) in the spleen, on day 8 p.i. of sporozoites and day 6 p.i. of iRBCs. Splenocytes isolated from infected mice and naïve controls were incubated with antigen presenting cells that were previously pulsed with Pb1 peptide. Pooled data with spot counts for each individual mouse are represented with median in the graph. Statistical significance was determined by One-way ANOVA with Bonferonni's correction or Kruskal Wallis with Dunn's multiple comparison (***p<0.0001, ** p<0.001, ns; not significant).
